# Rational construction of genome-reduced and high-efficient industrial *Streptomyces chassis* based on multiple comparative genomic approaches

**DOI:** 10.1186/s12934-019-1055-7

**Published:** 2019-01-28

**Authors:** Qing-Ting Bu, Pin Yu, Jue Wang, Zi-Yue Li, Xin-Ai Chen, Xu-Ming Mao, Yong-Quan Li

**Affiliations:** 1 Institute of Pharmaceutical Biotechnology & First Affiliated Hospital, Zhejiang University School of Medicine, Hangzhou, 310058 China; 2 Zhejiang Provincial Key Laboratory for Microbial Biochemistry and Metabolic Engineering, Hangzhou, 310058 China

**Keywords:** *Streptomyces chattanoogensis*, Cell factory, Computational approaches, Cre/*loxP* recombination system, Biological performances, Genome-reduced *chassis*, Secondary metabolites

## Abstract

**Background:**

*Streptomyces chattanoogensis* L10 is the industrial producer of natamycin and has been proved a highly efficient host for diverse natural products. It has an enormous potential to be developed as a versatile cell factory for production of heterologous secondary metabolites. Here we developed a genome-reduced industrial *Streptomyces chassis* by rational ‘design-build-test’ pipeline.

**Results:**

To identify candidate large non-essential genomic regions accurately and design large deletion rationally, we performed genome analyses of *S. chattanoogensis* L10 by multiple computational approaches, optimized Cre/*loxP* recombination system for high-efficient large deletion and constructed a series of universal suicide plasmids for rapid *loxP* or *loxP* mutant sites inserting into genome. Subsequently, two genome-streamlined mutants, designated *S. chattanoogensis* L320 and L321, were rationally constructed by depletion of 1.3 Mb and 0.7 Mb non-essential genomic regions, respectively. Furthermore, several biological performances like growth cycle, secondary metabolite profile, hyphae morphological engineering, intracellular energy (ATP) and reducing power (NADPH/NADP^+^) levels, transformation efficiency, genetic stability, productivity of heterologous proteins and secondary metabolite were systematically evaluated. Finally, our results revealed that L321 could serve as an efficient *chassis* for the production of polyketides.

**Conclusions:**

Here we developed the combined strategy of multiple computational approaches and site-specific recombination system to rationally construct genome-reduced *Streptomyces* hosts with high efficiency. Moreover, a genome-reduced industrial *Streptomyces chassis* *S. chattanoogensis* L321 was rationally constructed by the strategy, and the *chassis* exhibited several emergent and excellent performances for heterologous expression of secondary metabolite. The strategy could be widely applied in other *Streptomyces* to generate miscellaneous and versatile *chassis* with minimized genome. These *chassis* can not only serve as cell factories for high-efficient production of valuable polyketides, but also will provide great support for the upgrade of microbial pharmaceutical industry and drug discovery.

**Electronic supplementary material:**

The online version of this article (10.1186/s12934-019-1055-7) contains supplementary material, which is available to authorized users.

## Background

To date, more than 22,000 kinds of bioactive compounds from microbes were separated and described, over 45% of which were derived from *Actinomycetes*, particularly *Streptomyces*. A tremendous number of secondary metabolites produced by *Streptomyces* were utilized as lead compounds in medicine such as clinically important anticancer, antibiotic, anti-inflammatory, antiviral, anti-parasitic, antioxidant and anti-malaria drugs [1–4]. However, the production of many microbial drugs was very low in original strains because of the limitations of natural evolution. Meanwhile, genome analysis revealed that about 20–40 biosynthesis gene clusters were distributed in each *Streptomyces*. However, most of these gene clusters were cryptic under standard laboratory conditions. Therefore, in fact, *Streptomyces* was a huge natural reservoir of secondary metabolites and it was quite necessary to implement secondary prospecting of *Streptomyces* to discover more novel drugs against multidrug-resistant bacteria [5]. However, lots of *Streptomyces* were quite difficult to cultivate or even uncultivable under laboratory conditions, grown slowly, lacked efficient genetic manipulation, possessed complicated regulatory networks or produced a large number of endogenous by-products which impeded the progress of drug discovery [6, 7].

Heterologous expression is an efficient method to improve the production of microbial drugs and trigger the cryptic gene clusters for drug discovery [8]. The most important aspect of heterologous expression was to choose an efficient *chassis*. Although many kinds of microorganisms like *Escherichia coli* [9], *Bacillus subtilis* [10], *Pseudomonas putida* [11] could be utilized as *chassis*, a very large number of secondary metabolic gene clusters derived from *Streptomyces* could not or barely express in the *chassis* mentioned above because of the codon bias (high GC content) or unfitness of intrinsic regulatory networks or precursors [12, 13]. To date, only several *Streptomyces* had been developed as *chassis* like *Streptomyces coelicolor* A3(2), *Streptomyces avermitilis* MA-4680, *Streptomyces albus* J1074, *Streptomyces lividans* TK24. However, there still existed many heterologous gene clusters that could not be activated or barely expressed in available *Streptomyces chassis* [13, 14]. Therefore, it was quite necessary to develop novel or even universal high-performance *Streptomyces chassis* to increase the yield of well-known drugs and accelerate bioprospecting of diverse microbial resources.

With the development of high-throughput genome sequencing technology, a vast amount of complete genome sequences were available. Numerous researchers set about dissecting the functions of genomes and sophisticated cellular networks by comparative or functional genomics [15–17]. In 2005, the concept of pan-genome composed of core genome and dispensable genome that encompassed the complete repertoire of genes was proposed [18]. In the evolutionary context, the core genome mainly contained highly conserved genes in each individual genome, however the dispensable or accessory genome consisted of genes present in two or more but not all genomes and strain-specific genes. From the perspective of functional genomics, the vast majority of essential genes responsible for the basic functions of cell viability such as replication, transcription, translation, energy metabolism, cell division, ribosomal structure and biogenesis, distributed mainly in the core genome region, and the dispensable genome region was devoted to adaptation, antibiotic resistance, cell movement, virulence, transposition, secondary metabolites, which were non-essential to maintain normal cellular functions [19, 20]. Therefore, we assumed that the dispensable genome region can be deleted theoretically which may improve the performances of cells. According to the above hypothesis, construction of simplified or minimized genome by deleting non-essential genes based on systematic genome analyses would be feasible. A representative example of genome-minimized *Streptomyces* was the *S. avermitilis* SUKAs harboring 1.5 Mb-deletion which were constructed by Cre/*loxP*-mediated large deletion based on comparative genomics [21]. Recently, a cluster-free *S. albus chassis* was generated by deleting 15 endogenous biosynthetic gene clusters (BGCs) based on antiSMASH analysis [22]. A *S. coelicolor chassis* was also constructed by deleting 4 endogenous BGCs and ribosome engineering [23]. However, there still remain most of the non-essential elements like genome islands (GIs), insertion sequences (ISs) and BGCs in these *chassis* genomes which may result in genome instability or metabolic burden. Meanwhile, construction of *chassis* by one-by-one deletion of endogenous BGCs was time-consuming and laborious. Besides, current methods of large-scale genome editing only based on comparative genomics are too blindfold to precisely predict essential genes, and there still exist no systematic methods to analyze large-scale non-essential regions accurately which seriously hinder the process to develop high-efficient *chassis*.

Here, we developed a combinatorial strategy based on comparative, functional and pan-genomics to rationally design non-essential regions for construction of genome-reduced *chassis*. Meanwhile, the properties of many *Streptomyces chassis* were only evaluated by heterologous expression of BGCs or simplification of metabolic background which is too one-sided to reveal the intrinsic connection between genome streamline and emergent properties. Here, we firstly performed a systematic characterization of industrial *Streptomyces chassis* by phenotype changes, metabolic profiles, genetic stability, transformation efficiency, intracellular energy and reducing power, capability of protein expression and ability of heterologous expression of BGCs. This systematic evaluation will help us decipher the intrinsic relationships between genome reduction and the improved production of secondary metabolites.

*Streptomyces chattanoogensis* L10 is the industrial producer of natamycin (Type I PKS) and has been proved a highly efficient host for the production of diverse natural products [24, 25]. Since it was mainly responsible for PKS natural products, it has an enormous potential to be developed as a versatile cell factory for production of polyketides. Here, we applied our strategy to rationally construct and systematically evaluate the genome-reduced *S. chattanoogensis chassis* based on multiple genome analyses. Our strategy could not only be widely applied in other *Streptomyces* to generate more miscellaneous and versatile *chassis* with minimized genome, and to accelerate the development of synthetic biology, but also help us understand the underlying mechanism between genome reduction and enhanced performances.

## Results

### Determination of dispensable genetic elements

The complete genome sequence of *S. chattanoogensis* L10 has been determined by Roche 454 GS FLX [26, 27]. RAST server was used to perform genome annotation and KEGG analysis.

**Fig. 1:**
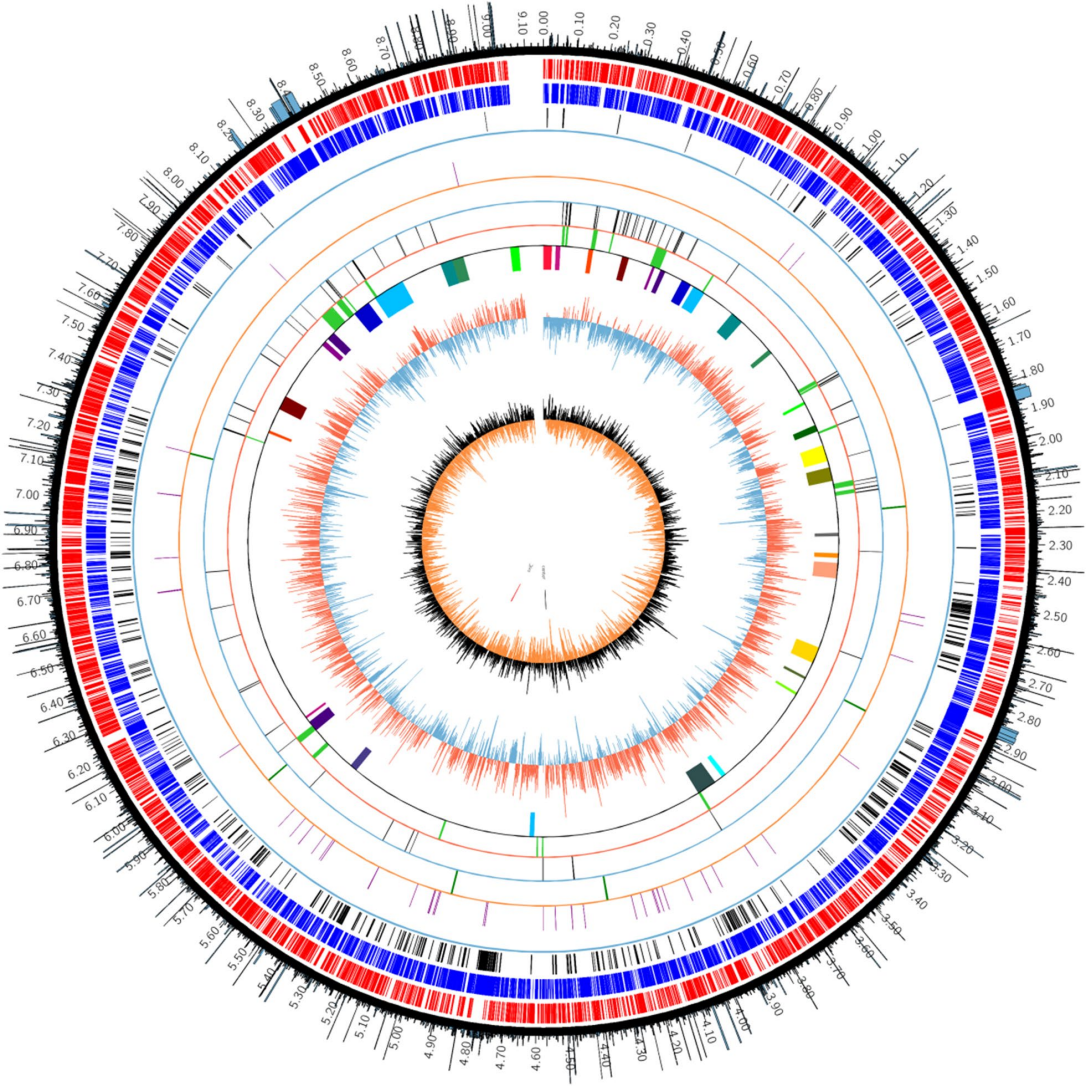
Circular map of *S. chattanoogensis* L10 genome. Circles 1 and 2 display the CDSs on the forward strand (red) and reverse strand (blue), respectively. Circle 3 displays the known essential genes. Circle 4 displays the tRNA genes. Circle 5 displays the rRNA genes. Circle 6 displays the ISs. Circle 7 displays the GIs. Circle 8 displays the BGCs. Circle 9 displays the GC percentage plot (±). Circle 10 displays the GC skew (±). The origin of replication is marked *oriC*. The center of chromosome is marked center. The outer scale is numbered in intervals of 0.1 Mbp. The outermost circle shows the ratios of essential genes. The genome map was made using Circos

Many studies had suggested that mobile genetic elements (MGEs), genomic islands (GIs) and biosynthesis gene clusters (BGCs) were dispensable. So we firstly performed analysis of dispensable components by computational approaches like antiSMASH [28], IslandViewer 4 [29], and ISsaga2 [30]. The results indicated that there were about 34 biosynthesis gene clusters (BGCs), 20 genomic islands (GIs) and 105 IS elements, and most of them were located at the two sub-telomeric regions. A type I-E CRISPR/*Cas* system was found at 8,084,591–8,095,933 bp by CRISPRfinder. It may decrease the efficiency of transformation or conjugation by targeting and degrading exogenous plasmids. The information of genome annotation and distribution of dispensable elements were visualized in the circular map of genome by Circos (Fig. 1). We can clearly see that these dispensable elements concentrated in both side of genome (0–3.0 Mb and 7.4–9.0 Mb). However, we cannot yet determine the boundary of non-essential regions because some essential genes may distribute at both sides. Locations of these redundant elements would help us to determine the candidate target deletion regions more rationally.

### Pan-genome and comparative genome analyses

In order to determine the boundary of non-essential regions accurately, we need to investigate the functions and distributions of essential genes. Based on the assumption that essential genes were highly conserved during the process of evolution, we performed comparative analysis of five *Streptomyces* complete proteomes, *S. albus* J1074, *S. avermitilis* MA-4680, *S. chattanoogensis* L10, *S. coelicolor* A3(2) and *S. griseus* by OrthoVenn [31]. The results suggested that about 2702 proteins were highly conserved in all the five *Streptomyces* proteomes, and 973 of them were function-unknown and others were mainly responsible for basic cellular functions like DNA replication, transcription, translation, ribosomal biogenesis and primary metabolism.

**Fig. 2:**
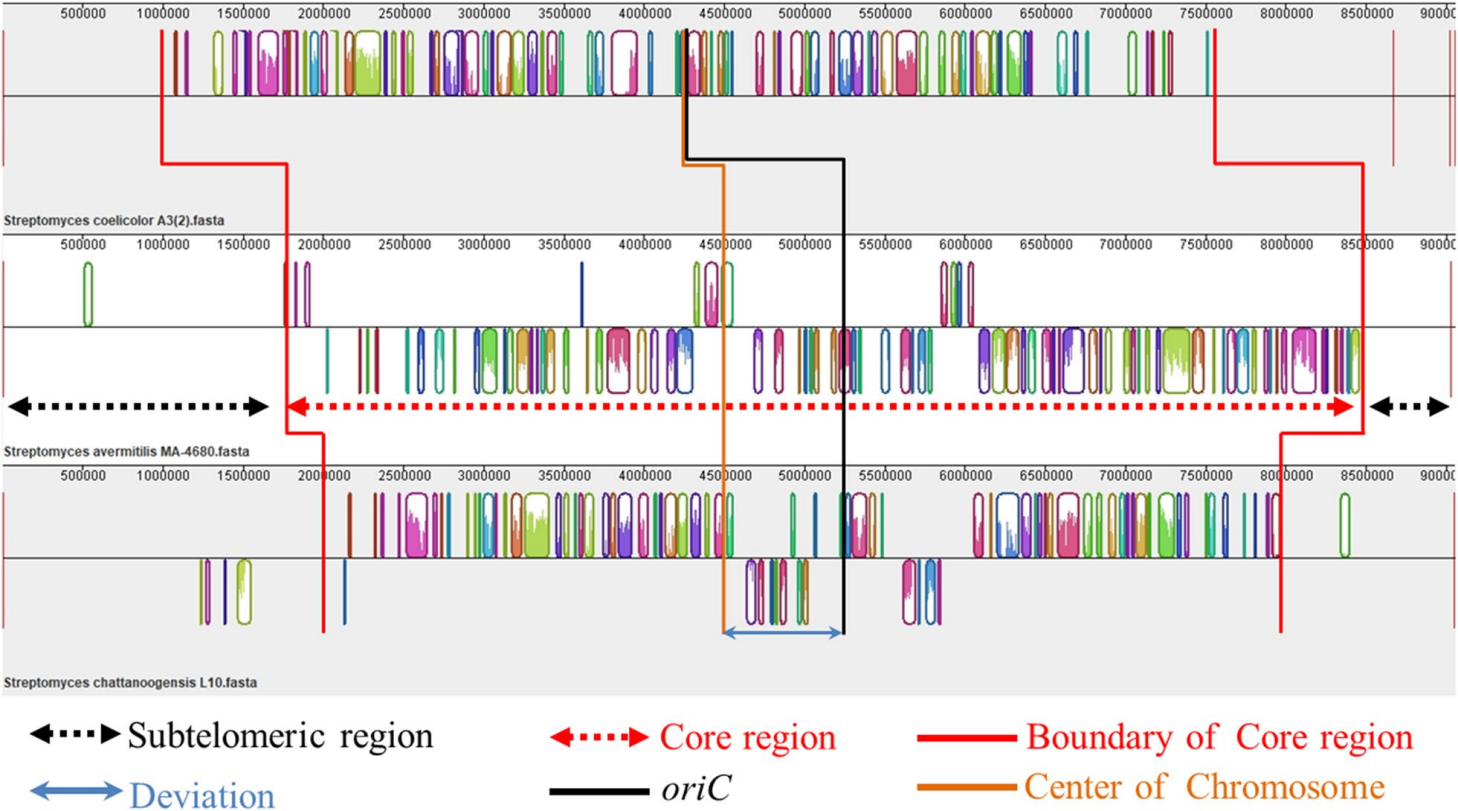
Multiple genome alignments by Mauve 2.3.1. Comparative analysis of three taxonomically distinct *Streptomyces* genomes, *S. avermitilis* MA-4680, *S. coelicolor* A3(2), and *S. chattanoogensis* L10, revealed a conserved core region of ∼ 6.0 Mb in which the majority of the genes are highly conserved with a high degree of synteny and two dispensable (accessory) genome regions located at sub-telomeric regions of the chromosomal ends. The *oriC* and *dnaA* box-like sequences are located at 5,293,751–5,294,960 bp which has about a deviation of 770 Kb to the center of chromosome, so the genome structure shows asymmetry

In order to determine the functions and distributions of putative essential genes, we performed pan-genome and comparative genome analyses by Bacterial Pan Genome Analysis pipeline (BPGA) [32] and Mauve 2.3.1 [33]. The results revealed that the 9 Mb-size genome of *S. chattanoogensis* L10 consisted of about 6.0 Mb core genomic region around the origin of replication (*oriC*) with symmetry, and 2.0 Mb and 1.0 Mb dispensable (accessory) genome regions located at sub-telomeric regions of the chromosomal ends, respectively. Meanwhile, pan-genome analysis suggested that approximate 2650 genes are present in all individuals, which was virtually consistent with the OrthoVenn results. And the KEGG function analysis indicated that the two dispensable genome regions are mainly composed of non-essential genes like secondary metabolite-associated genes, strain-specific genes, transposition-associated genes which were not necessary for primary metabolism and robust cellular functions, and can be deleted theoretically. Local blast analysis revealed that the *oriC* and *dnaA* box-like sequences were located at 5,293,751–5,294,960 bp which had about a deviation of 770 Kb to the center of chromosome. So the genome structure was asymmetric which indicated that the two non-essential regions may be also different in size (Fig. 2).

Besides, we also submitted the whole-genome sequence to DEG 10 (Database of Essential Genes) [34] and ARTS (Antibiotic Resistant Target Seeker) [35] for determination of known essential and duplicated genes. The results indicated that about 589 genes were classified as essential genes in which 65 genes showed duplication. We focused on these duplicated genes located at each side of genome in which one can be deleted without affecting another. And we also found that some essential genes and their duplications appeared in 0–0.5 Mb and 8.7–9.0 Mb regions, respectively, which indicated that the two regions cannot be removed at the same time. Therefore, previously described two dispensable regions (0–3.0 Mb and 7.4–9.0 Mb) were reduced as 0.5–3 Mb and 7.4–8.7 Mb. We further analyzed other well-known essential genes and found that a large number of essential genes distributed in 7.5–7.8 Mb and 1.9–2.75 Mb. Finally, we chose the 0.5–1.9 Mb and 8.0–8.7 Mb regions as candidate non-essential regions. The ratios of essential genes were also shown in the circular map of genome outermost circle with histogram (Fig. 1).

Finally, in overall view of the distributions of non-essential elements and essential genes, and the results from comparative genomics and pan-genomics analyses, two candidate genomic regions were deemed to be removable. The two non-essential regions were about 1.3 Mb and 0.7 Mb-size located at 499,650–1,841,266 bp and 7,994,797–8,731,201 bp, respectively. Subsequently, we attempted to delete the two candidate regions by Cre/*loxP* recombination system.

### Optimization of Cre/*loxP* recombination system

Although Mamoru Komatsu et al. had successfully developed thiostrepton-induced Cre/*loxP* system in *S. avermitilis*, the thiostrepton was highly toxic to *S. chattanoogensis* L10. So we optimized the Cre/*loxP* system by replacing the thiostrepton-induced promoter *tipAp* with PnitA-NitR system named pNitCre which was inducible by ε-caprolactam. Meanwhile, based on pSET152 plasmid, we constructed a series of universal suicide vectors containing *loxP* or mutant *loxP* (*loxP66* or *loxP71*) sites which can be inserted into genome by single crossover.

In order to determine whether Cre enzyme can work normally, pSATDF was introduced into *S. chattanoogensis* L10. After Cre enzyme expression, we identified 48 clones randomly by PCR and confirmed that recombination has taken place between two *loxP* sites in all selected clones by sequencing PCR products. The results suggested that Cre enzyme can work in *S. chattanoogensis* L10 with high efficiency and a new *loxP* site was formed. When we performed deletion of an 80 Kb *trans*-AT PKS gene cluster, the precise deletion of the targeted gene cluster was also observed with high frequency. The schematic diagram showed the procedure of pSATDF and pSATPR integrated into genome by homologous recombination and Cre-mediated site specific recombination (Additional file 1).

**Fig. 3:**
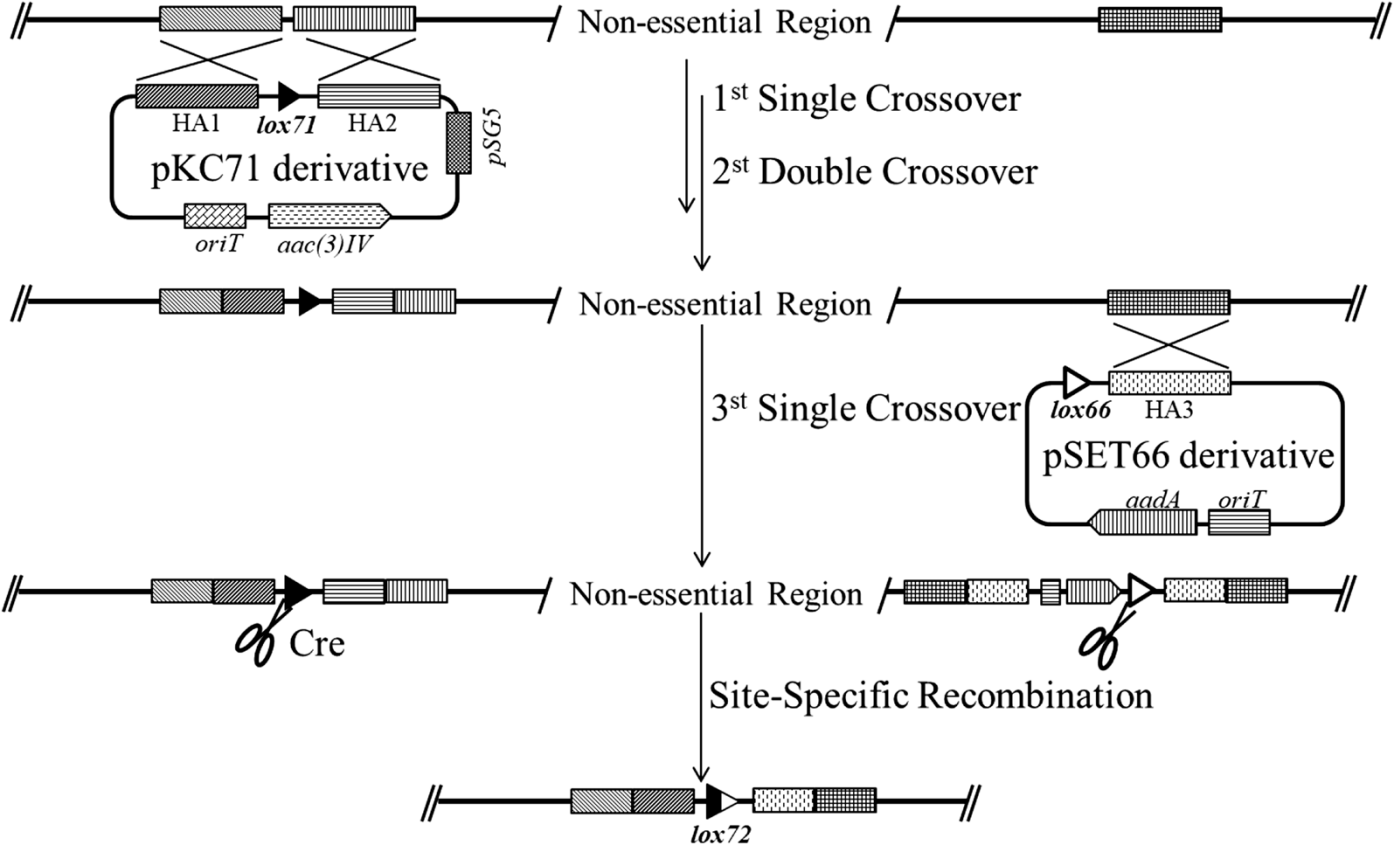
Strategy for construction of large-deletion mutants by optimized Cre/*loxP* system. Two nonessential gene regions (499,650–1,841,266 bp, 7,994,797–8,731,201 bp) are selected as candidate targeted deletion regions based on comparative genomic analysis. *LoxP* mutant site *lox71* is introduced into genome by pKC1139-mediated double crossover. Another *loxP* mutant site *lox66* is introduced into genome by suicide vector-mediated single crossover. Expression of Cre enzyme is induced by 0.1% ε-caprolactam to mediate site-specific recombination between *lox71* and *lox66* after pNitCre is introduced

Subsequently, we chose to delete the two large dispensable genome regions. We introduced two mutant *loxP* sites with the same orientation flanking the two regions individually (Fig. 3). Replica plating method was used to identify the mutants. We can see that all clones can growth on YMG plate without antibiotics but not on corresponding plate with spectinomycin (Additional file 2). The above results showed that site-specific recombination has taken place between the *lox71* and *lox66* site. PCR and sequencing had proven that the two large non-essential gene regions had been deleted. Finally, we successfully obtained the mutants with large deletions, 1.3 Mb and 0.7 Mb, named L320 and L321, respectively. However, when we tried to combine the two large deletions, mutant with combinatorial deletion could not be screened which indicated that the two large regions could not be deleted synchronously. We proposed that maybe some paired or complementary orthologous genes located in the two large regions functionally complement deletion of each other, therefore they could not be deleted simultaneously. Therefore, we performed functional analysis of every putative essential gene which was predicted by DEG (Database of essential genes) and found that some paired essential genes with same function were located at the two targeted regions (1.3 Mb and 0.7 Mb) separately. These paired essential genes like urease-associated genes (*orf1441* and *orf7491*, *orf1443* and *orf7492*, *orf1445* and *orf7494*), dTDP-4-dehydrorhamnose 3,5-epimerase encoding genes (*orf1209* and *orf7573*), glycerol kinase encoding genes (*orf2011* and *orf7622*) and peptide deformylase encoding genes (*orf1463* and *orf7856*) involved in amino acid transport and metabolism, cell envelope biogenesis, energy production and conversion, translation or ribosomal structure and biogenesis processes, respectively. These processes, in particular cell envelope and ribosomal biogenesis, were fundamental for a life which indicated that synchronous deletion of these gene pairs will be lethal. All of known essential genes with duplication were linked by Bézier curve in the circular genome map (Additional file 3).

Growth and development analysis of the two mutants revealed that there were no noticeable differences in the morphological development and sporulation processes on solid sporulation medium compared to their parental strain. However, the mutant L320 showed decreased growth rate and abnormal mycelial growth (data not shown) and L321 maintained similar biomasses in liquid medium compared with its ancestral strain (Additional file 4). Therefore, we finally chose L321 as the *chassis* to evaluate its performances for heterologous expression of proteins or biosynthetic gene clusters.

### Secondary metabolite profiles

**Fig. 4:**
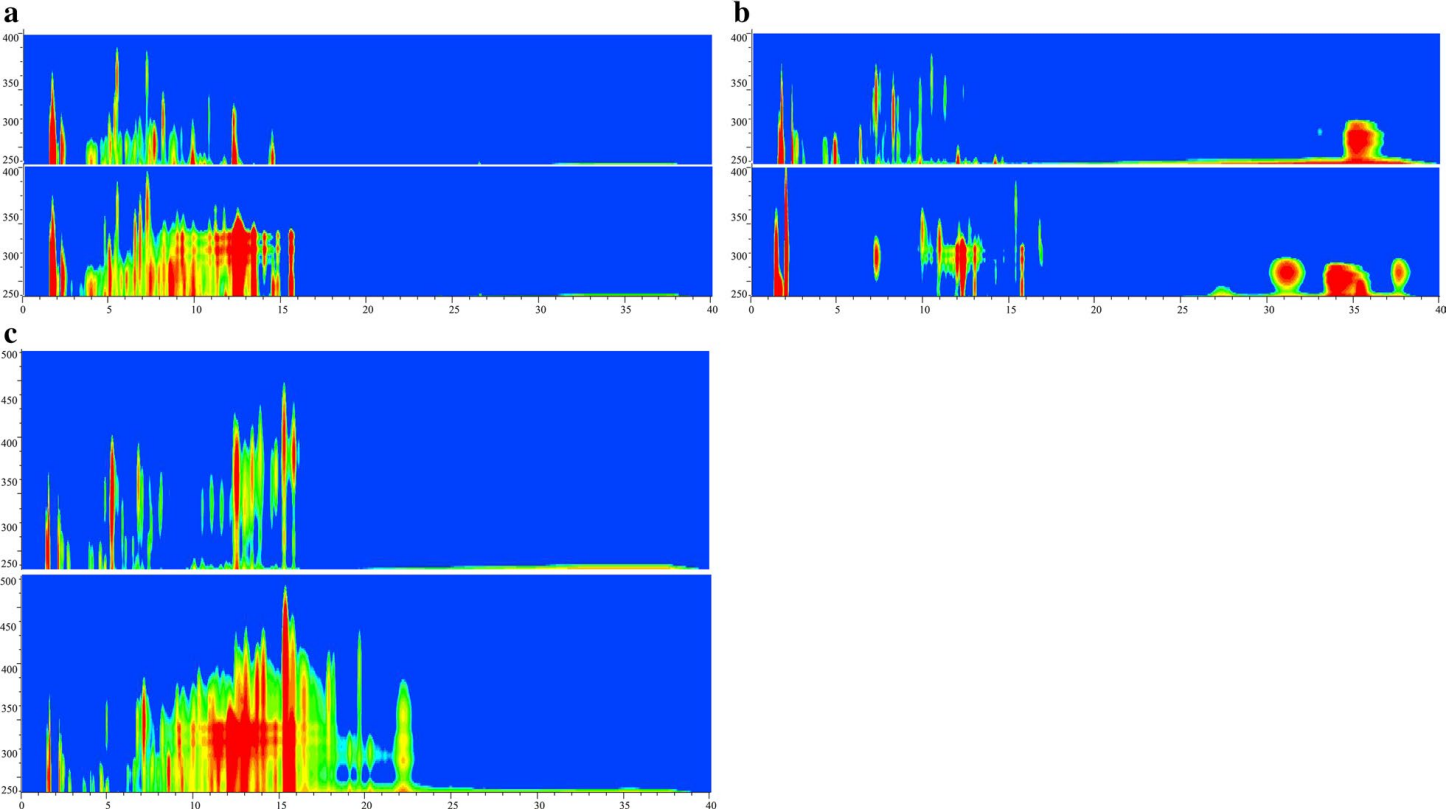
Metabolite profiles analysis based on iso-absorbance plot. *S. chattanoogensis* L10 (down) and L321 (up) are inoculated about 120 h in different fermentation media, YSG (**a**), YEME (**b**) and ISP2 (**c**). The methanol extract of fermentation broth is analyzed by HPLC with wavelength scanning from 190 nm to 600 nm. x axis represents HPLC time (min) and y axis represents absorption wavelength (nm)

L321 harbored 0.7 Mb deleted region consisting of 7 putative BGCs and complete natamycin biosynthetic gene cluster (the main product). Here, we firstly investigated the HPLC metabolite profiles of L321 in different fermentation media such as YEME, ISP2, YSG. By means of wavelength scanning and iso-absorbance plot analysis, we found that the main metabolite natamycin and many other products had disappeared in different media in L321 (Fig. 4). The results demonstrated that L321 possessed cleaner and simpler metabolite profiles than its parental strain which would lay a good foundation for heterologous expression of proteins or gene clusters.

### Productivity of heterologous proteins

**Fig. 5:**
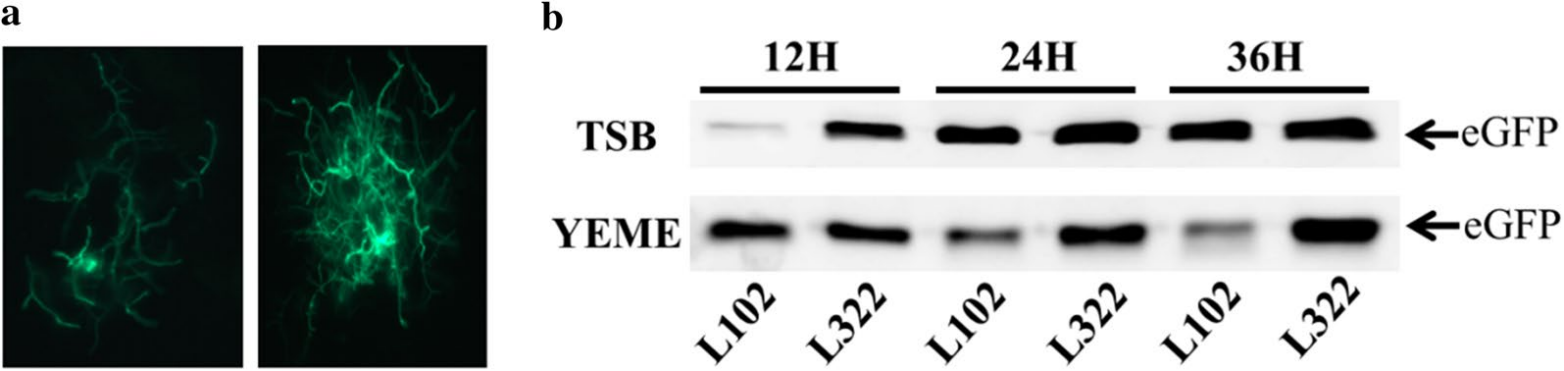
The expression levels of eGFP in *S. chattanoogensis* L102 and L322. **a** The expression of eGFP in L102 (left) and L322 (right) are observed by fluorescence microscope. The observed mycelia are sampled from YEME fermentation broth at 24 h. **b** eGFP are detected by Western blot. Two different media are selected. TSB medium was mainly used for vegetative mycelia growth, however, YEME medium was for triggering secondary metabolite biosynthesis. pIJ8668-*ermeP*-*egfp* is used to overexpress *egfp* gene in *Streptomyces*

**Fig. 6:**
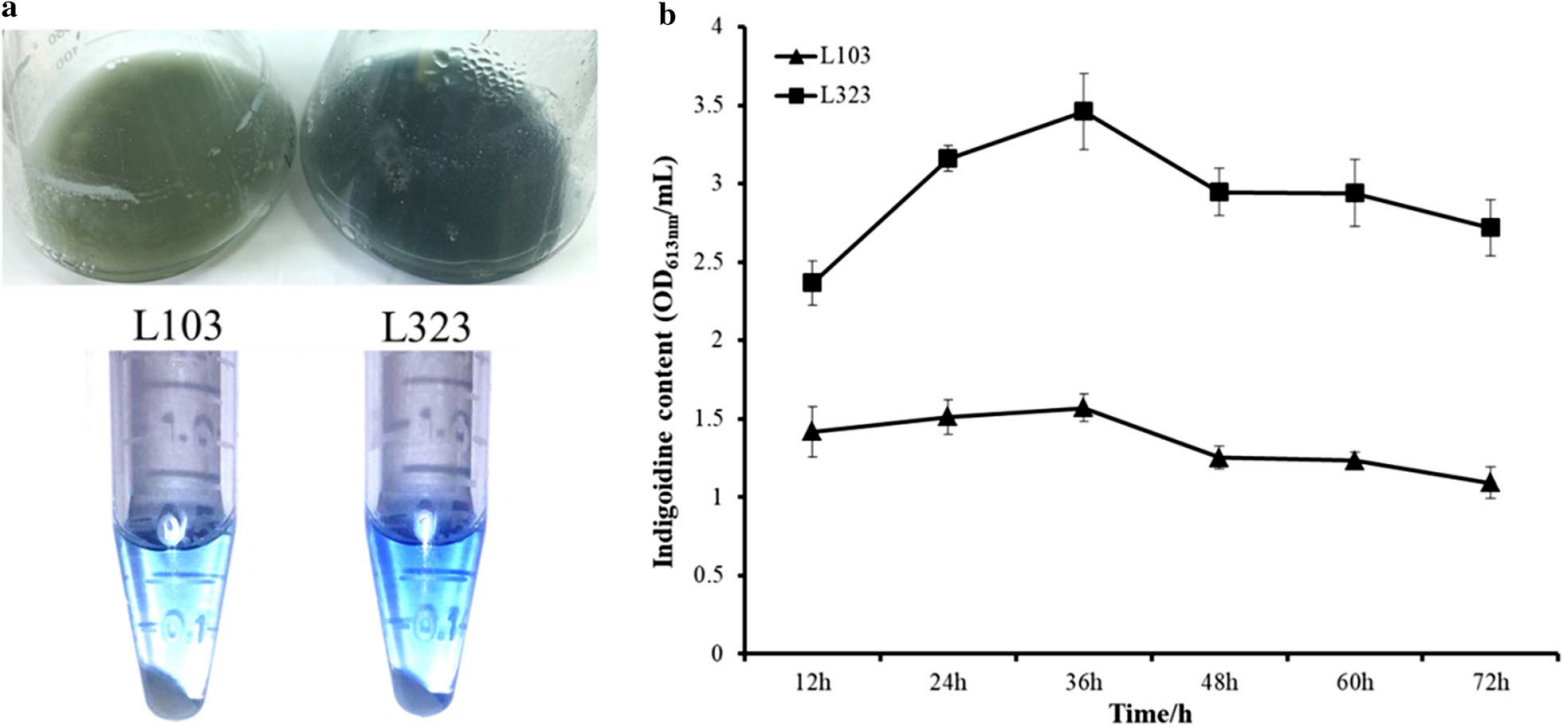
The expression of a single-module non-ribosomal peptide synthase (IndC) from *S.albus* J1074 in *S. chattanoogensis* L103 and L323. **a** Phenotype of L103 and L323 incubated in TSB medium about 36 h (Up) and blue pigment indigoidine extracted with DMSO from L103 to L323 mycelia (Down). **b** The indigoidine content from L103 to L323 per 1 mL fermentation broth was measured at several times. Error bars indicate SD of samples performed in triplicate

Subsequently, we investigated the productivity of eGFP in L321 and wild type L10 in different media, TSB and YEME. We introduced the eGFP expression plasmid pL100 into L10 and L321 to get L102 and L322, respectively. The expressions of eGFP in L102 and L322 were observed by fluorescence microscope (Fig. 5a). We can see that the expression of eGFP in L322 was enhanced compared with that in L102. The expression level of eGFP in the two media was measured at different times by Western blot. In TSB medium, the expression level of eGFP was gradually increased both in L102 and L322 but the concentration of eGFP was higher in L322, however, in YEME medium, eGFP was gradually degraded in L102 but enhanced in L322 from 12 to 36 h. The result suggested that the productivity of eGFP in L322 mutant was clearly higher and more stable than that in L102 (Fig. 5b). Besides, we also performed heterologous expression of a single-module non-ribosomal peptide synthase (IndC) which was responsible for the biosynthesis of a blue pigment indigoidine [36]. pTEindC was integrated into L10 and L321 to get L103 and L323. We can obviously see that the production of indigoidine was improved in L323 compared with that in L103 (Fig. 6a). The yields of indigoidine in L103 and L323 were measured at 613 nm. The results suggested that the production of indigoidine in L323 was 2–3 times higher than that in L103 (Fig. 6b). The above results indicated that L321 not only enhanced expression capability of heterologous proteins but also may have a potential to expression NRPS natural products or drugs.

### Intracellular energy and reducing power

**Fig. 7:**
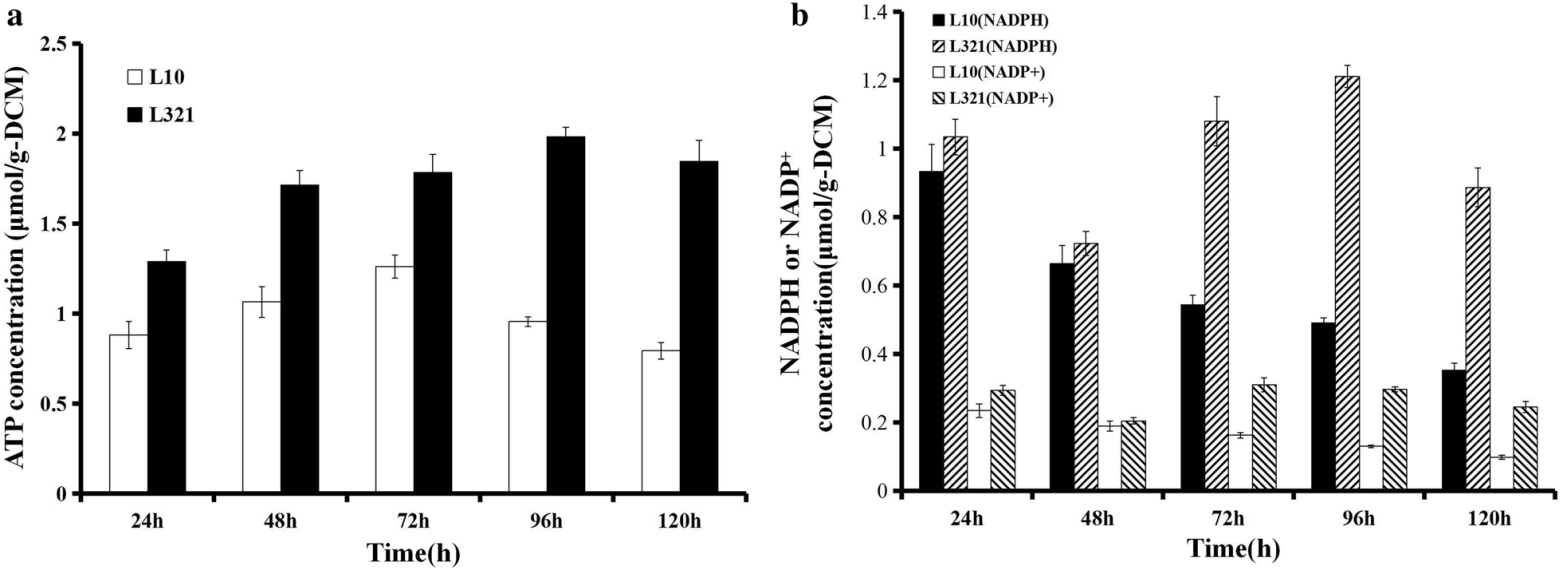
Intracellular ATP, NADPH and NADP^+^ concentrations. **a** Intracellular ATP concentration in *S. chattanoogensis* L10 and L321 at different times. **b** Intracellular NADPH and NADP^+^ concentrations in *S. chattanoogensis* L10 and L321 at different times. DCW, dry cell weight. Error bars indicate SD of samples performed in triplicate

Large deletion in L321 may economize more cellular energy (ATP) and reducing power (NADPH/NADP^+^) for improving the production of heterologous proteins. We investigated the intracellular ATP and NADPH/NADP^+^ levels in L321 and L10. The results suggested that the intracellular ATP and NADPH/NADP^+^ levels are higher in L321 than that in L10 (Fig. 7). The enhanced intracellular ATP and NADPH/NADP^+^ levels in L321 may contribute to the higher productivity of heterologous proteins.

### Transformation efficiency

**Fig. 8:**
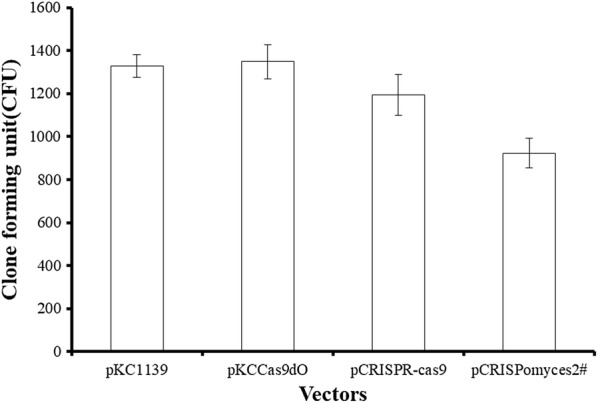
Transformation efficiency of several CRISPR/Cas systems in *S. chattanoogensis* L321. pKCCas9dO, pCRISPR-Cas9 and pCRISPomyces2# were several well-established CRISPR/Cas systems in *Streptomyces*, pKC1139 was used as negative control. These CRISPR/Cas systems cannot be introduced into *S. chattanoogensis* L10 (no transformants). The error bars represent standard deviations of the means of triplicate samples

We found that there were no obvious differences of transformation efficiency between L321 and L10 when pKC1139 and pSET152 were used (data not shown). However, when we tried to introduce CRISPR/Cas9 systems into *S. chattanoogensis* L10, we failed to obtain any transformants although we had optimized multiple conditions of conjugation. Meanwhile, we also failed to introduce CRISPR/Cas9 systems into other industrial *Streptomyces* in our lab like *S. tsukubaensis* YN06, *S. albus* ZD11. The Cas9 proteins maybe highly toxic to these industrial *Streptomyces* or their endogenous CRISPR/Cas systems may conflict with heterologous CRISPR/Cas9 or there existed other unknown reasons. Intriguingly, we can successfully introduce several CRISPR/Cas9 systems into L321. The efficiency of transformation of pCRISPR-Cas9 [37] and pKCCas9dO [38] in which the expression of Cas9 was induced by thiostrepton was approximately consistent with pKC1139, however, the efficiency decreased when pCRISPomyces [39] in which the Cas9 gene is under the control of constitutive promoter rpsLp(XC) was used (Fig. 8). In order to explain above phenomenon, we analyzed the endogenous CRISPR/Cas system by CRISPRfinder. The results showed there was an endogenous type I-E CRISPR/Cas system in L10 which has been deleted in L321. So we proposed that the endogenous CRISPR/Cas system may interfere with heterologous CRISPR/Cas9 systems, which decreased the transformation efficiency of CRISPR/Cas9 plasmids and restricted the extensive use of these systems in industrial *Streptomyces*. Therefore it is well worth investigating the intrinsic regulatory mechanisms in order to generalize these high efficient CRISPR/Cas9 systems in *Streptomyces* to improve the efficiency of genome editing.

### Productivity of secondary metabolite

**Fig. 9:**
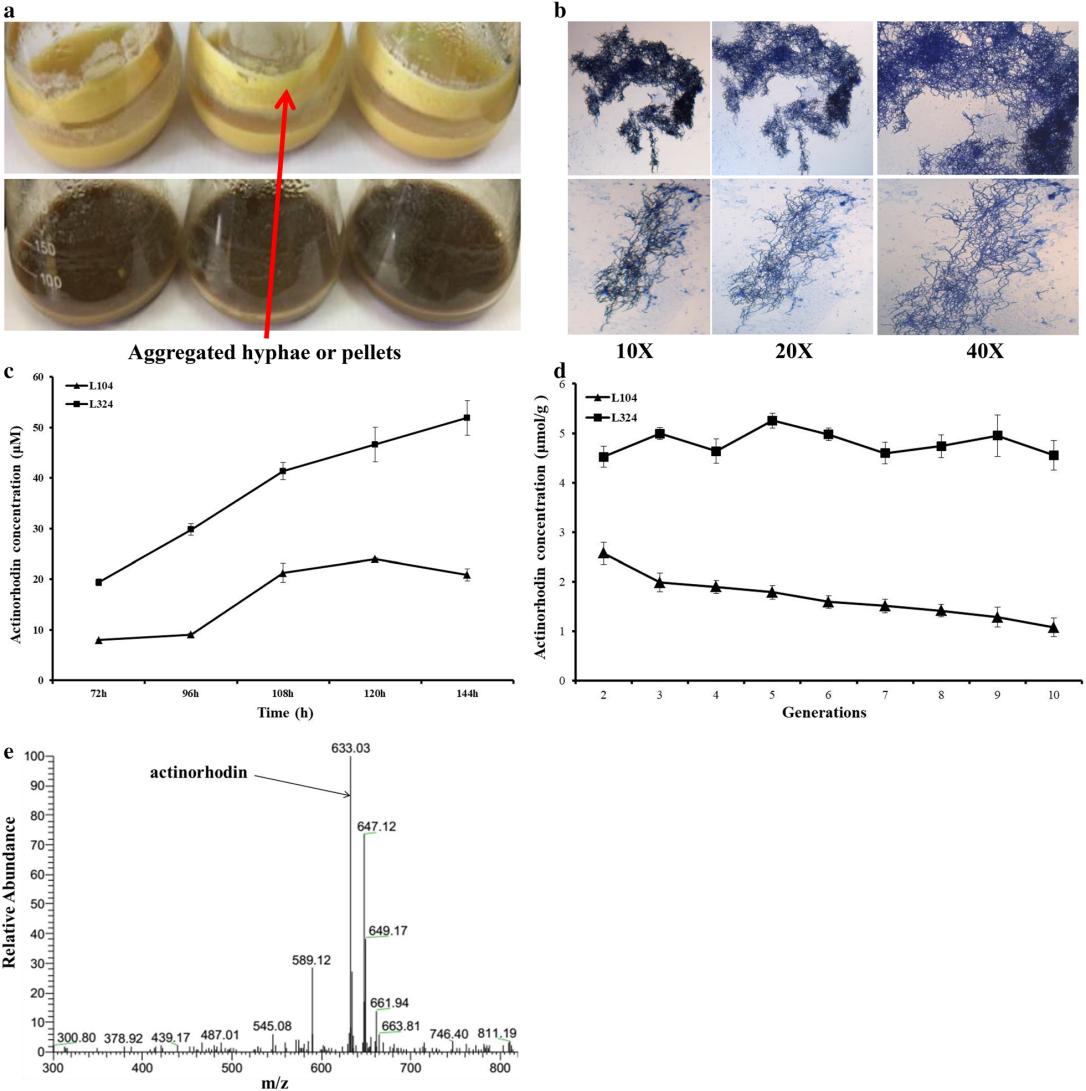
Morphological differentiation, actinorhodin production and genetic stability analyses. **a** Mycelia of L104 formed aggregated hyphae or noticeable pellets on glass wall, but L324 mutant cannot. (B) Mycelia were observed by microscope at magnifications 10×, 20×, and 40×. L104 and L324 were inoculated in YEME medium about 120 h. **c** The actinorhodin production in L104 and L324 were measured at different times. **d** Genetic stability of L104 and L324. Strains are performed serial passaging on YMG plate and actinorhodin production in YMG plate was determined after 10 days incubation. The error bars represent standard deviations of the means of triplicate samples. **e** LC–MS analysis of actinorhodin in negative ion mode from metabolites of L104 and L324

Since L321 improved the productivity of heterologous proteins, the biosynthesis of heterologous BGCs may also be enhanced. We introduced the pMM1 harboring actinorhodin gene cluster into L10 and L321 to obtain L104 and L324, respectively. Actinorhodin is a well-characterized pH indicator (red/blue) metabolite. As we can easily see, the productivity of actinorhodin in L324 was higher than that in L104. Besides, we noticed that during the fermentation process, L10 derivative L104 gradually congregated to form lots of pellets around the wall of flask, but L321 derivative L324 cannot (Fig. 9a). We also observed mycelial morphology by microscope at magnifications 10×, 20×, and 40× and found that the mycelia of L324 were more dispersed than those of L104 (Fig. 9b). This morphological change may be advantageous for industrialization because dispersed mycelia were better to assimilate oxygen and nutriment, and we will probe into this emergent phenotype in ‘Discussion’ section. Meanwhile, we also measured the concentration of actinorhodin by UV spectrophotometry, and the results suggested that the yield of actinorhodin in L324 was 2–3 times higher than that in L104 (Fig. 9c).

### Genetic stability

As a platform cell, the genome stability is also vital for expression of natural products. Mobile genetic elements like insertion sequences (ISs) are the important factors resulting in genome instability [40]. IS-mediated mutagenesis and genomic rearrangements will result in instability of strains harboring engineered genes or gene clusters which may inactivate genes or decrease the production of secondary metabolites. In L321, about 15% of putative insertion sequences (ISs) were deleted. We investigated the stability of actinorhodin in L104 and L324 by serial passaging on YMG plate. After 10 generations, the actinorhodin of every generation in YMG plate was extracted by 1 N KOH and measured by UV spectrophotometry. The production of actinorhodin was rather stable in L324 but was gradually lost in L104 (Fig. 9d). The results indicated that deletion of insertion sequences (ISs) in L321 may decrease IS-mediated random mutagenesis and increase its genetic stability. Besides, the actinorhodin in L104 and L324 was determined by LC–MS in negative ion mode (Fig. 9e).

## Discussion

Although comparative genomic analysis has been successfully used to predict the putative non-essential genes in *S. avermitilis*, it is difficult to determine the reducible regions accurately only by genome alignment. To identify redundant genes more reliably and design deletions more rationally, we further performed pan-genome and functional genome analyses by BPGA and OrthoVenn on the basis of genome alignment. The results revealed that 9 Mb-size genome of *S. chattanoogensis* L10 consisted of about 6 Mb core region and 2.0 Mb and 1.0 Mb dispensable (accessory) regions located at sub-telomeric regions of the chromosomal ends, respectively. The core region mainly contained about 2700 essential genes which were conserved during the evolutionary process and some function-unknown genes. However, the dispensable regions were mainly composed of non-essential genes which were not necessary for primary metabolism and robust cellular functions and can be deleted theoretically. Meanwhile, we analyzed GIs, ISs, BGCs and CRISPR/Cas system which were deemed to be unnecessary and underlying candidate genes to be deleted for further genome reduction. In order to determine the boundary of non-essential regions, we investigated the functions and distributions of known essential genes by DEG10 and ARTS. In order to avoid synthetic lethality, we paid more attention to duplications of these essential genes. Finally, we took all these factors into account to determine two candidate non-essential regions which were deleted successfully. Here, we developed combinatorial strategy based on comparative genomics and functional genomics to systematically analyze genome and rationally determine non-essential genomic regions. This strategy can be widely applied in other microorganisms to analyze large redundant regions for constructing genome minimized *chassis*, especially industrial *chassis* to accelerate the process of biomedicine industrialization.

We also optimized the Cre/*loxP* system for deleting large non-essential genomic regions efficiently. Previously we have tried to perform large deletion with pALCre in which the *cre* gene is under control of *tipAp* promoter but failed. Due to the relatively high background expression of inducible promoter *tipAp* and toxicity of thiostrepton (tsr) to some *Streptomyces*, we optimized the tsr-induced Cre/*loxP* recombination system by PnitA-NitR. PnitA-NitR system has been proved a hyper-inducible expression system for *Streptomyces* which is tightly controlled by ε-caprolactam. Meanwhile, ε-caprolactam is an inexpensive and non-toxic inducer which can be widely used [41]. In this study, we constructed pNitCre based on pL99 [42] to control the expression of Cre enzyme and a series of universal suicide plasmids based on pSET152 convenient for *loxP* or *loxP* mutant sites inserting into genomes. Finally, we developed the combined strategy of multiple computational approaches and site-specific recombination system to rationally construct genome-reduced hosts with high efficiency, and successfully constructed two genome-reduced *Streptomyces* hosts L320 and L321 harboring 1.3 Mb and 0.7 Mb genomic deletions, respectively. The above results proved the feasibility of our strategy.

Unfortunately, the two large dispensable regions cannot be combined into a single combinatorial deletion. This phenomenon indicated that pairs of unknown genes located in the two large non-essential regions maybe have synthetic lethal effects with each other so that cannot be deleted simultaneously, which also termed synthetic lethality. Many synthetic lethality analyses like SGA, dSLAM, E-MAP, RNAi also have been developed to study pairs of synthetic lethal genes in *E. coli, S. cerevisiae, Caenorhabditis elegans* [43, 44]. Similar strategies can be performed to determine the functions of synthetic lethal genes in *Streptomyces* which will provide crucial reference for rational construction of genome-minimized hosts, especially by combinatorial deletion technique. Moreover, according to the results of IslandViewer, ISsaga2, antiSMASH, deletion of partial non-essential genes (GIs, ISs, BGCs) dispersed in core region in L321 mutant can further simplify the genome and may further improve its biological performances as cell factory.

Unexpectedly, the L321 formed dispersed hyphae morphology in liquid YEME medium while the L10 congregated a large number of pellets around the wall of flask (Fig. 7a). After methylene blue staining, the hyphae morphology was observed by microscope. We can see that the mycelia of L321 were more dispersed, and the mycelia of L10 had aggregated to form large number of mycelial pellets (Fig. 7b). Many studies have shown that the adhered mycelia formed aggregated hyphae or pellets on glass wall which resulted in deterioration of oxygen and nutrient delivery to the inner aggregated hyphae. And the aggregated hyphae will seriously restrict the further application in industrialization [45]. Therefore, morphological engineering has been performed to improve the production of secondary metabolites in *Streptomyces*. Many morphogenes like *ssgA, cslA, matAB* or *glxA* have been genetically engineered to inhibit pellet formation and promote the production of antibiotics. SsgA protein played positive roles in inhibit pellet formation, however, CslA and MatAB proteins can stimulate the hyphae aggregation to form pellets [46–48]. In L321, large deletion may have an effect on the expression of these morphogenesis which resulted in more fragmented or dispersed hyphae. Therefore, the homologues of *ssgA, cslA, matAB* morphogenesis were found by local blast against L10 genome and the transcription level of these genes were determined by qRT-PCR. And the results suggested that the transcription level of *ssgA* was enhanced at 48 h when the hyphae began to aggregate and the transcription level of *cslA* was decreased seriously in L321 at all times, however, the transcription level of *matAB* was down-regulated in stationary phase when a large number of megascopic pellets had adhered to the wall of flask in L10 (Additional file 5). These changes of expression level of morphogenes in L321 may have a significant effect on its morphology. Further experiments could be performed to explore intrinsic molecular mechanism which is of great benefit to industrialization.

**Fig. 10:**
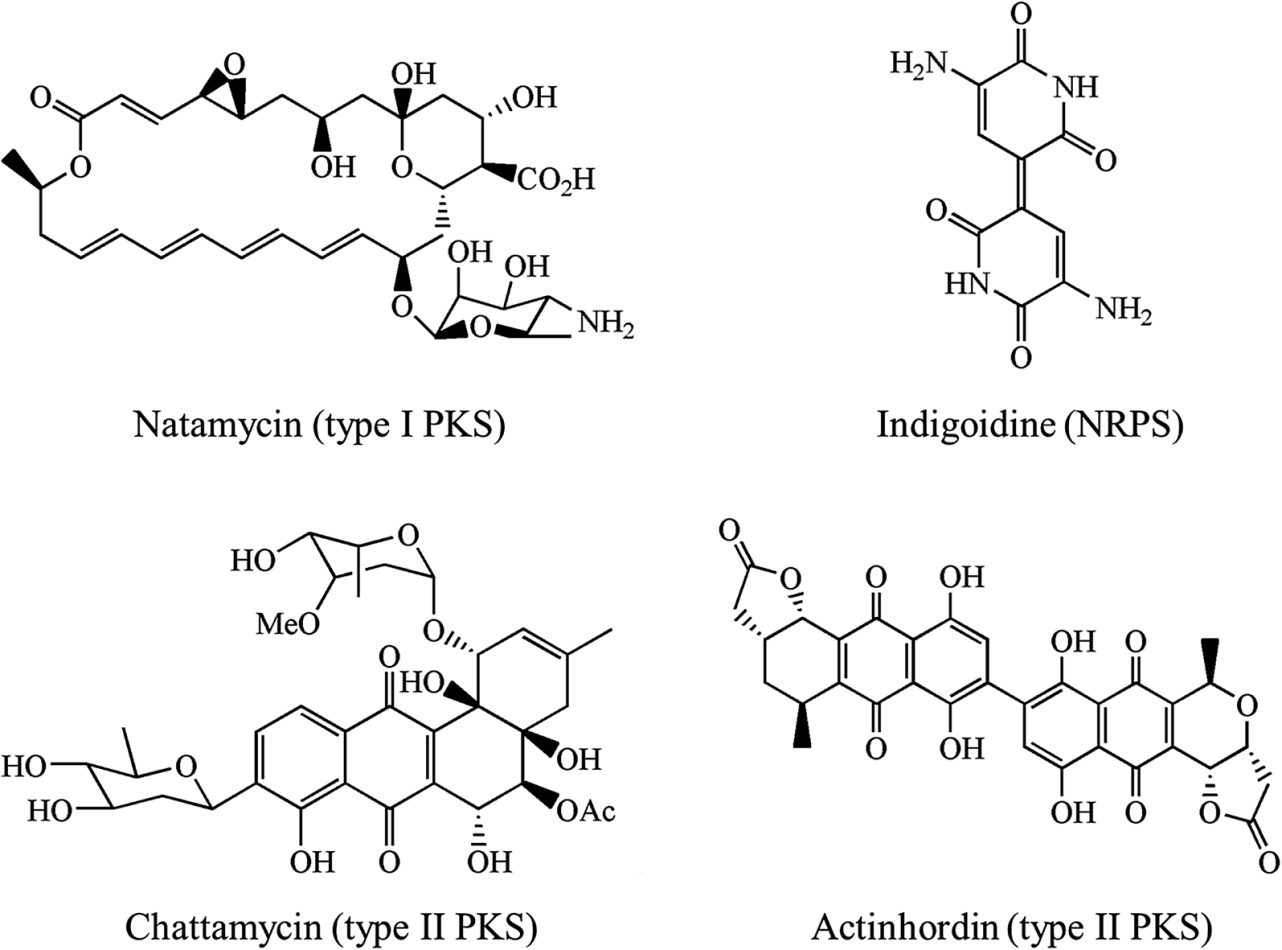
Chemical structures of natamycin, chattamycin, actinorhodin and indigoidine

Finally, we proposed that large-scale genome reduction can not only eliminate the interference of by-products or non-targeted metabolites, but also enrich metabolic fluxes like primary metabolic fluxes into targeted pathways to enhance the production of natural products or microbial drugs. Different types of secondary biosynthetic pathways may depend on different primary metabolic processes like glycolysis, TCA cycle, amino acids metabolism, pentose phosphate pathway for supplying different precursors, energy, reducing power or cofactors. *S. chattanoogensis* L10 is the industrial producer of natamycin and it also can produce a large number of chattamycin A-B. Natamycin and chattamycin belong to polyketides. So *S. chattanoogensis* can supply plenty of polyketide’s precursors like malonyl-CoA (M-CoA), methylmalonyl-CoA (MM-CoA) to secondary metabolic pathways, especially PKS pathways. We also successfully performed heterologous expression of a type II PKS gene cluster (actinorhodin). Besides, we also expressed a single-module non-ribosomal peptide synthase (IndC) for indigoidine which indicated that L321 has a potential to synthesize NRPSs. The chemical structures of natamycin, chattamycin, actinorhodin and indigoidine were shown as follows (Fig. 10). L321 also exhibited several emergent and excellent performances for heterologous expression of secondary metabolite, like enhanced intracellular energy (ATP) and reducing power (NADPH/NADP^+^), improved productivity of proteins and secondary metabolite, more dispersed mycelia, increased transformation efficiency, simplified metabolite profiles, increased genetic stability. Therefore, the genome-minimized industrial *Streptomyces chassis* L321 can serve as a promising cell factory of actinorhodin. In our future study, we will perform heterologous expression of PKS gene clusters, especially cryptic and unknown gene clusters, to improve the production of well-known drugs or to excavate novel products by activating silent gene clusters. Besides, the influences of genome reduction on metabolic fluxes can be revealed by isotope-labeled metabolic flux analysis (MFA) which will be probed in our future research.

## Conclusion

To identify candidate large non-essential genomic regions accurately and design large deletion rationally, we performed systematic genome analyses by multiple computational approaches, optimized Cre/*loxP* recombination system for high-efficient large deletion and constructed a series of universal suicide plasmid for rapid *loxP* or mutant *loxP* sites inserting into *Streptomyces* genome. Moreover, a genome-reduced industrial *Streptomyces chassis* L321 was rationally constructed by the combined strategy, and the *chassis* exhibited several emergent and excellent performances for heterologous expression of secondary metabolite, like enhanced intracellular energy (ATP) and reducing power (NADPH/NADP^+^), improved productivity of protein and secondary metabolite, more dispersed mycelia, increased transformation efficiency, simplified metabolite profiles, increased genetic stability. We proposed that the *chassis* L321 can serve as a promising platform cell to produce polyketides. We expected that this combined strategy could be widely applied in other *Streptomyces* to generate more miscellaneous and versatile *chassis* with minimized genome. These *chassis* not only can serve as cell factories for high-efficient production of valuable microbial drugs, even plant drugs, but also will provide great support for the upgrade of microbial pharmaceutical industry and drug discovery.

## Methods

### Bacterial strains and growth conditions

Plasmids and strains used in this study were listed in Table S2 **(**Additional file 6) and Table S3 (Additional file 7). *E. coli* TG1 was used as host for plasmid construction. *E. coli* DH10B was used for the propagation of large plasmid DNA. *E. coli* ET12567/pUZ8002 was used as donor for intergeneric conjugation to *S. chattanoogensis* L10 or its mutants. *E. coli* was grown in liquid Luria–Bertani medium (1% tryptone, 0.5% yeast extract, and 1% NaCl) at 37 °C on a rotary shaker at 220 rpm. The components of media for *S. chattanoogensis* were as follows, YEME(0.3% yeast extract, 0.3% malt extract, 0.5% tryptone, 4% glucose); ISP2 (0.4% yeast extract, 1.0% malt extract, 0.4% glucose, 0.2% CaCO3 pH 7.2 ~ 7.3); YSG (2.8% soybean flour, 0.7% yeast extract, 6% glucose); TSB(3% TSB). Solid medium contained 2% agar. *S. chattanoogensis* L10 or mutant strains sporulation, growth and genome DNA preparations were carried out as described previously [24]. If needed, antibiotics or inducer were supplemented to growth media at the following concentrations: 100 μg/mL ampicillin, 50 μg/mL apramycin, 100 μg/mL spectinomycin, 25 μg/mL chloramphenicol, 50 μg/mL kanamycin, 30 μg/mL nalidixic acid or 0.1% ε-caprolactam.

### DNA manipulations and cloning

HiPure Gel Pure DNA Mini Kit (Magen) was used to purify PCR fragments and Plasmid Miniprep Kit (Zoman) was used to isolate plasmid DNA. All the restriction enzymes, alkaline phosphatase, T4 DNA ligase and DNA marker were purchased from Thermo. High-fidelity PCRs and general PCRs were performed with KOD-Plus-Neo (TOYOBO) and 2 × Hieff™ PCR Master Mix (YEASEN), respectively.

### Plasmid construction and intergeneric conjugation

All the primers used in this study were listed in Table 4 (Additional file 8). Primers were synthesized by GENEray (Shanghai, China). pSET152 plasmid was digested by restriction enzyme *Hin*dIII and self-ligated by T4 DNA ligase to obtain the suicide vector pSET153. Primer pairs aadA-F/aadA-R was used to amplify the aadA resistance gene from pIJ779 and ligated into pSET153 by SacI to get pSET154. The fragment containing *loxP* site was amplified by primer pairs *loxP*-F1/*loxP*-R1 and *loxP*-F2/*loxP*-R2 with the plasmid pUG66 as template, and digested with *Hin*dIII/BglII and EcoRV/EcoRI, then ligated with same restriction enzymes digested pSET154 to get the plasmid pSETD. pUG66 was digested with *Hin*dIII and XbaI to get another restriction fragment containing *loxP* site. Then the restriction fragment was ligated into the *Hin*dIII and XbaI sites of pSET154 to get the plasmid pSETP. Primer pairs lox66-F/lox66-R and lox71-F/lox71-R were denatured for 5 min at 95 °C and anneal at 4 °C, and ligated into pSET154 and pKC1139 respectively to get pSET66 and pKC71. The schematic diagrams of universal plasmids pSETD, pSETP, pSET66 and pKC71 were shown in Additional file 9. Primer pairs ATD-F/ATD-R were used to amplify a 3 Kb homologous fragment from the genomic DNA, the PCR products were ligated into pTA2 and sequenced, then the plasmid was digested with XbaI and BglII. The restriction fragment was ligated with pSETD to get pSATDF. Primer pairs ATP-F/ATP-R are used to amplify another 2.6 Kb homologous fragment which was ligated into pSETP by EcoRV and EcoRI to get pSATPR. Primer pairs LR-F/LR-R and RR-F/RR-R were used to amplify 2 Kb homologous fragments, the two homologous fragments were ligated into pSET66 by EcoRV/EcoRI to get pSLR and pSRR. Primer pairs LF-F1/LF-R1, LF-F2/LF-R2, RF-F1/RF-R1 and RF-F2/RF-R2 were used to amplify corresponding homologous arms which were ligated into pKC71 by corresponding restriction sites to get pKCLF and pKCRF. Primer pairs Cre-F/Cre-R were used to amplify the *cre* gene with the plasmid pALCre as template. The PCR products were ligated into pTA2 and sequenced. Then the right plasmids were digested and ligated into pL99 by NdeI/BamHI to get the plasmid pNitCre. ermE promoter were amplified by primer pairs ermEp-F/ermEp-R from pL97 to ligated into pTOS by *Hin*dIII/SpeI to get pTOSE. Primer pairs indC-F/indC-R were used to amplify indC gene from *S. albus* J1074 genome and the indC gene was ligated into pTOSE by NdeI/XbaI sites to get plasmid pTEindC. All plasmids were sequenced before transformed into the conjugation donor *E. coli* ET12567/pUZ8002.

pSATDF was introduced into *S. chattanoogensis* L10 by conjugation and exconjugants were selected on YMG plate supplement with spectinomycin and identified by PCR. Then pNitCre was conjugated into the exconjugants and the expression of Cre enzyme was induced by 0.1% ε-caprolactam. The induced mixture was screened by replica plating. In order to delete an 80 Kb *trans*-AT PKS gene cluster located at 1,808,377–1,888,379 bp, we inserted another loxP site by pSATPR with same method. After induced with 0.1% ε-caprolactam for 10 h, the induced mixture was screened by replica plating. For deletion of non-essential regions, we introduced two mutant *loxP* sites with the same orientation flanking the two regions individually. *Lox71* site was introduced by double crossover based on pKC71 derivative pKCLF and pKCRF, and lox66 was inserted by suicide vector-mediated single crossover based on plasmid pSET66 derivative pSLR and pSRR. pNitCre was introduced into the two mutants and 0.1% ε-caprolactam was used to induce the expression of Cre enzyme. The replica plating method and PCR were utilized to identify the mutants. Recombinant plasmids were introduced into *S. chattanoogensis* by intergeneric conjugation as previously described [24].

### Genome analysis software and application

RAST (Rapid Annotation using Subsystem Technology) (http://rast.nmpdr.org/) [49] was used to analyze and annotate the sequenced genomes.

Basic Local Alignment Search Tool (ftp://ftp.ncbi.nlm.nih.gov/blast/executables/blast+/2.8.0alpha/) was a command-line version on Windows system which was used to quickly align nucleotide or protein sequences with target genome.

AntiSMASH (Antibiotics and Secondary Metabolite Analysis Shell) bacterial version (https://antismash.secondarymetabolites.org/) was used to predict the secondary metabolite biosynthesis gene clusters of sequenced genomes.

IslandViewer 4 (http://www.pathogenomics.sfu.ca/islandviewer/) was used to predict the large genomic islands that are thought to be from horizontal gene transfer.

ISsaga2 (http://issaga.biotoul.fr/issaga_index.php) was used to predict the insert sequences (ISs).

CRISPRfinder (http://crispr.i2bc.paris-saclay.fr/Server/) was used to analyze the putative endogenous CRISPR/Cas system.

OrthoVenn (http://www.bioinfogenome.net/OrthoVenn/start.php) was used to align all proteins and predict the highly conserved proteins among several strains.

BPGA (Bacterial Pan Genome Analysis pipeline) (http://www.iicb.res.in/bpga/index.html) was used to analyze the core genome and dispensable genome.

Mauve 2.3.1 (http://darlinglab.org/mauve/mauve.html) was used to construct and visualize multiple genome alignments.

DoriC (http://tubic.tju.edu.cn/doric/index.php) was a database of bacterial and archaeal replication origins used to analyze and locate the origin of replication (oriC).

DNAMAN (https://www.lynnon.com/dnaman.html) was used to align nucleotide or protein sequences.

DEG10 (http://www.essentialgene.org) was a database of essential genes used to predict known essential genes in annotated genome.

ARTS (Antibiotic Resistant Target Seeker) (https://arts.ziemertlab.com) was an exploration engine for antibiotic cluster prioritization and novel drug target discovery which can be used to predict secondary metabolite biosynthesis gene clusters and also can be used to analyze the duplication of essential genes.

Circos (http://circos.ca/) [50] was used to visualize the distribution of genes, BGCs, GIs, ISs.

### HPLC metabolite profiles

The fermentation broth from YEME, YMG or YSG was extracted with an equal volume of methanol and centrifuged 10 min at 12000 rpm. The supernatant was filtered through 0.45-μm membrane. 20 μL of the supernatant was injected into the Agilent 1260 HPLC system and wavelength scanning was performed between 190 nm and 600 nm. ZORBAX Eclipse XDB C18 was used as the column, H_2_O (containing 0.1% formic acid) and acetonitrile (containing 0.1% formic acid) were used as the mobile phase A and B performing a linear gradient from 5 to 95% (v/v) B over 35 min, with a subsequent isocratic stage of 95% B for 5 min. The column was further equilibrated with 5% B for 5 min and the flow rate was 1 mL/min. The analysis of metabolite profiles was performed by iso-absorbance plot which presented the wavelength, time and peak intensity as a contour map.

### Detection of actinorhodin

To measure the production of actinorhodin in solid medium, the same amount of spores were spread on YMG plate and incubated about 10 days at 30 °C, then top of 1 mL pipette tip was used to scratch cylindrical medium which was put into 900 μL 1 N KOH. The mixture was subjected to 3 freeze–thaw cycles between − 80 °C and 25 °C, centrifuged at 12000 rpm for 10 min. To determine the concentration of actinorhodin in liquid medium, 1 mL fermentation broth was treated with 500 μL 3 N KOH, vortexed thoroughly and centrifuged at 4000×*g* for 10 min. The absorption of the supernatant was determined at λ640 nm. Actinorhodin concentration was calculated based on the Lambert–Beer’s law using molar extinction coefficient of ε640 = 25,320 that corresponds to pure actinorhodin. In order to perform LC–MS analysis of actinorhodin, L104 and L324 were incubated in YEME medium at 30 °C for 120 h. Fermentation broths were adjusted to pH 2–3 with 2 M HCl. Acidified fermentation broths were extracted with triple volume EtOAc and evaporated under reduced pressure. The extract was re-suspended with 200 μL of methanol. LC–MS analysis was performed in an Agilent 1200 HPLC system (Agilent, Santa Clara, CA, United States) and a Thermo Finnigan LCQDeca XP Max LC/MS system (Thermo Finnigan, Waltham, MA, United States). ZORBAX Eclipse XDB C18 was used as the column, H_2_O (containing 0.1% formic acid) and acetonitrile (containing 0.1% formic acid) were used as the mobile phase A and B performing a linear gradient from 20 to 100% (v/v) B over 35 min.

### Measurement of indigoidine

UV spectrophotometry was used to determine the content of indigoidine according to previously described method [36] with some modifications. In brief, 500 μL fermentation broth was centrifuged at 12,000 rpm for 10 min and the supernatant was removed. The mycelia were washed twice with PBS buffer. Pellets were re-suspended in 500 μL DMSO and sonicated (on 3 s, off 3 s, 3 times) with 30% power. The samples were centrifuged at 12,000 rpm for 10 min and the supernatants were transferred to a 1.5 mL sterile EP tube. The absorption of the supernatant was determined at λ_613 nm_. All experiments were performed in triplicate.

### Western Blot

The expression level of eGFP in L102 and L322 was determined by Western Blot as described previously [42]. Briefly, spores were inoculated into 35 mL of seed medium for 20 h in a 250 mL flask. Then cell density (OD_600nm_) was determined by UV spectrophotometry. Seed culture was transferred into 35 mL of TSB medium in a 250 mL flask, set up with a starting OD_600nm_ of 0.15. After incubation at 30 °C for 12 h, 24 h, 36 h, 48 h in a rotary shaker, 500 μL mycelia were collected and washed once with 1 mL PBS buffer, finally re-suspended in 500 μL PBS buffer. The mycelia suspensions were sonicated on ice (4 × 5 s, with 5 s intervals every time). The samples were centrifuged at 12,000 rpm for 10 min at 4 °C and the supernatants were transferred to a 1.5 mL sterile EP tube. The total protein quantification was performed by Bradford assay. Then 15 μg of total protein were separated in 12% SDS-PAGE and western blot analysis was performed with rabbit polyclonal anti-EGFP antibody (Proteintech, USA).

### Total RNA isolation and qRT-PCR

The total RNA of *S. chattanoogensis* L10 and its mutants was prepared with EASYspin Plus bacteria RNA extract kit (Aidlab) according to the manufacturer’s instructions. The residual genomic DNA was removed by RNase-free DNase I (Takara). The cDNA was prepared using PrimeScript™ 1st Strand cDNA Synthesis Kit (Takara) according to the manufacturer’s instructions. Quantitative real-time PCR was performed on Roche LightCycler 480 (Roche) with the SYBR Premix Ex Taq (Takara) in 20 μL volume according to the manufacturer’s instructions. The expression level of *hrdB* was used as the internal reference. All values were normalized to the corresponding expression level of *hrdB* and all experiments were performed in triplicate.

### Measurement of intracellular ATP, NADPH and NADP^+^

In order to determine the concentrations of intracellular ATP, NADPH and NADP^+^, *S. chattanoogensis* L10 and its mutants were incubated in YEME medium. The mycelium were collected by centrifugation and washed twice with PBS buffer. The concentrations of intracellular ATP, NADPH and NADP^+^ were measured by ATP measurement kit and NADP(H) measurement kit (Solarbio) according to the manufacturer’s protocols. And the biomass or dry cell weight (DCM) was also measured. The concentrations were calculated by measured content (μmol)/DCM (g). All experiments were performed in triplicate.

## Additional files

**Additional file 1.** The schematic diagram shows pSATDF and pSATPR integrated into genome by homologous recombination and Cre-mediated site specific recombination.

**Additional file 2.** The replica plating method to screen mutants with non-essential region deletion. Left plates are without antibiotics, right plates are supplemented with 100 μg/mL spectinomycin. Clones 1–13 represent the 1.3 Mb deletion, clones 14–26 represent 0.7 Mb deletion. (A) Front side of replica plates (B) Back side of replica plates.

**Additional file 3.** All of known essential genes with duplication were linked by Bézier curve in the circular genome map.

**Additional file 4.** Growth curves of *S. chattanoogensis* L10 and L321. Biomasses (dry cell weight per 1 mL fermentation broth) are measured in different times with 12 h interval. Error bars indicate SD of samples performed in triplicate.

**Additional file 5.** Relative mRNA expression level of morphogenesis in *S. chattanoogensis* L10 and L321. Transcription analysis of morphogenesis *ssgA, clsA* and *matAB* was carried out by qRT-PCR. *clsA* gene barely expressed, so the data were not shown. The transcription of sigma factor *hrdB* gene was assessed as an internal control. The expression level of theses morphogenesis in L10 is set as 1. The error bars represent standard deviations of the means of triplicate samples.

**Additional file 6.** Table S2 shows plasmids used in this study and short description.

**Additional file 7.** Table S3 shows strains used in this study and short description.

**Additional file 8.** Table S4 shows primers used in this study and short description.

**Additional file 9.** Schematic diagrams of universal plasmids pSETD, pSETP, pSET66 **(A)** and pKC71 **(B)**.

## Abbreviations

PKS: polyketide synthase
NRPS: non-ribosomal peptide synthase
antiSMASH: antibiotic and secondary metabolite analysis shell
IS: insertion sequence
DEG: Database of Essential Genes
DSB: double strands break
MGEs: mobile genetic elements
GIs: genome islands
BGCs: biosynthetic gene clusters
RAST: Rapid Annotation using Subsystem Technology
CRISPR: clustered regularly interspaced short palindromic repeats
BPGA: Bacterial Pan Genome Analysis pipeline
oriC: origin of replication
KEGG: Kyoto Encyclopedia of Genes and Genomes
ARTS: Antibiotic Resistant Target Seeker
MCS: multiple clone sites
HPLC: high performance liquid chromatography
eGFP: enhanced green fluorescent protein
DMSO: dimethyl sulfoxide
PBS: phosphate buffer solution
SDS-PAGE: sodium dodecyl sulfate-polyacrylamide gel electrophoresis
DCM: dry cell weight
RNAi: RNA interference
SGA: synthetic genetic array
dSLAM: heterozygote diploid-based synthetic lethality analysis with microarrays
E-MAPs: epistatic miniarray profiles
RT-PCR: real-time polymerase chain reaction
TCA: tricarboxylic acid cycle
MFA: metabolic flux analysis
