# Supplementary figures and images for: Rational construction of genome-reduced and high-efficient industrial Streptomyces chassis based on multiple comparative genomic approaches
Source: Microb Cell Fact. 2019 Jan 28;18:16. doi: 10.1186/s12934-019-1055-7 (PMC6348691; doi:10.1186/s12934-019-1055-7)

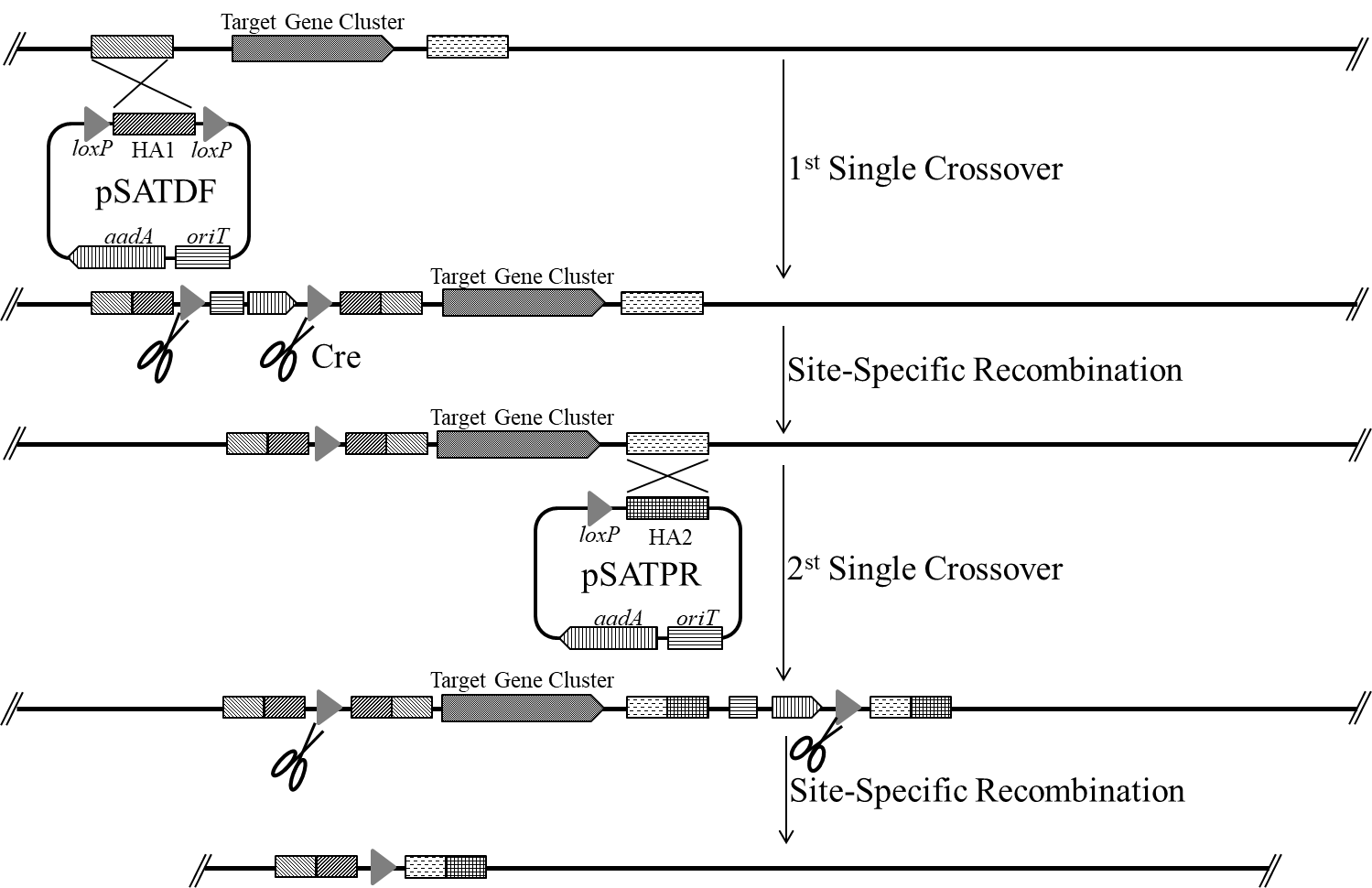

Supplement: Supplementary file 1 — Additional file 1. The schematic diagram shows pSATDF and pSATPR integrated into genome by homologous recombination and Cre-mediated site specific recombination. [file 12934_2019_1055_MOESM1_ESM.docx]

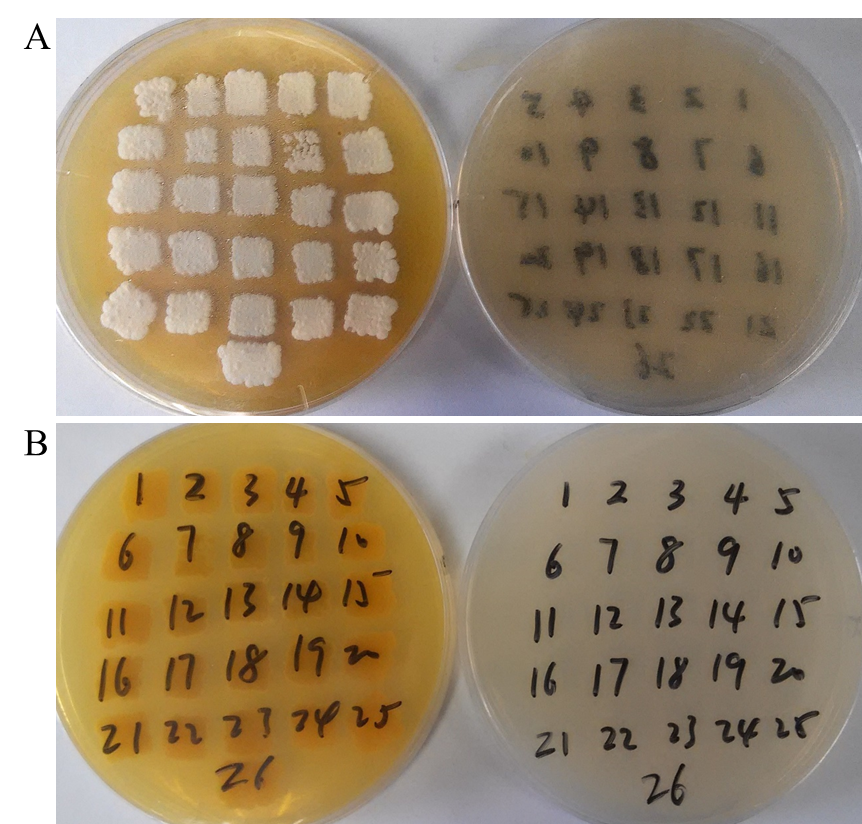

Supplement: Supplementary file 2 — Additional file 2. The replica plating method to screen mutants with non-essential region deletion. Left plates are without antibiotics, right plates are supplemented with 100 μg/mL spectinomycin. Clones 1–13 represent the 1.3 Mb deletion, clones 14–26 represent 0.7 Mb deletion. (A) Front side of replica plates (B) Back side of replica plates. [file 12934_2019_1055_MOESM2_ESM.docx]

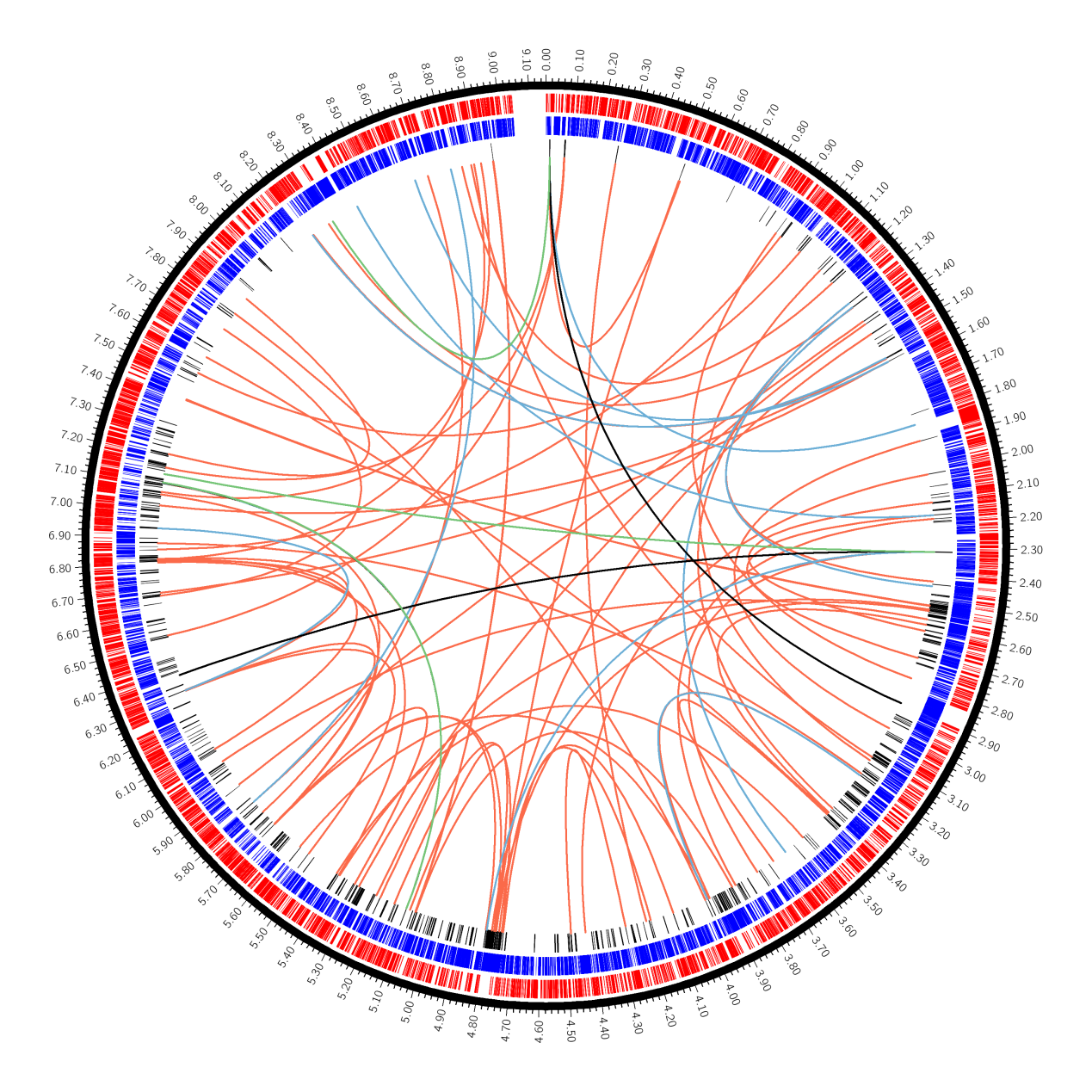

Supplement: Supplementary file 3 — Additional file 3. All of known essential genes with duplication were linked by Bézier curve in the circular genome map. [file 12934_2019_1055_MOESM3_ESM.docx]

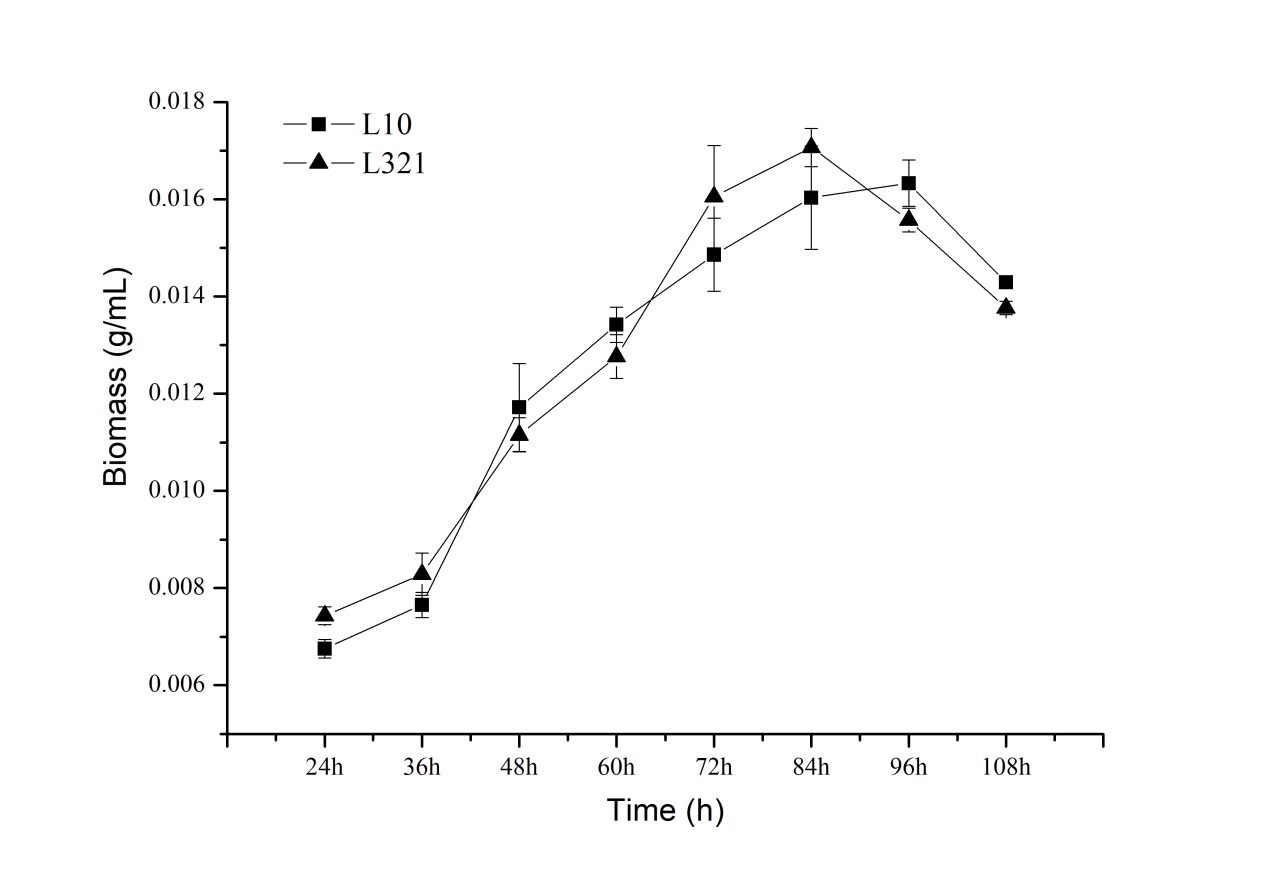

Supplement: Supplementary file 4 — Additional file 4. Growth curves of S. chattanoogensis L10 and L321. Biomasses (dry cell weight per 1 mL fermentation broth) are measured in different times with 12 h interval. Error bars indicate SD of samples performed in triplicate. [file 12934_2019_1055_MOESM4_ESM.docx]

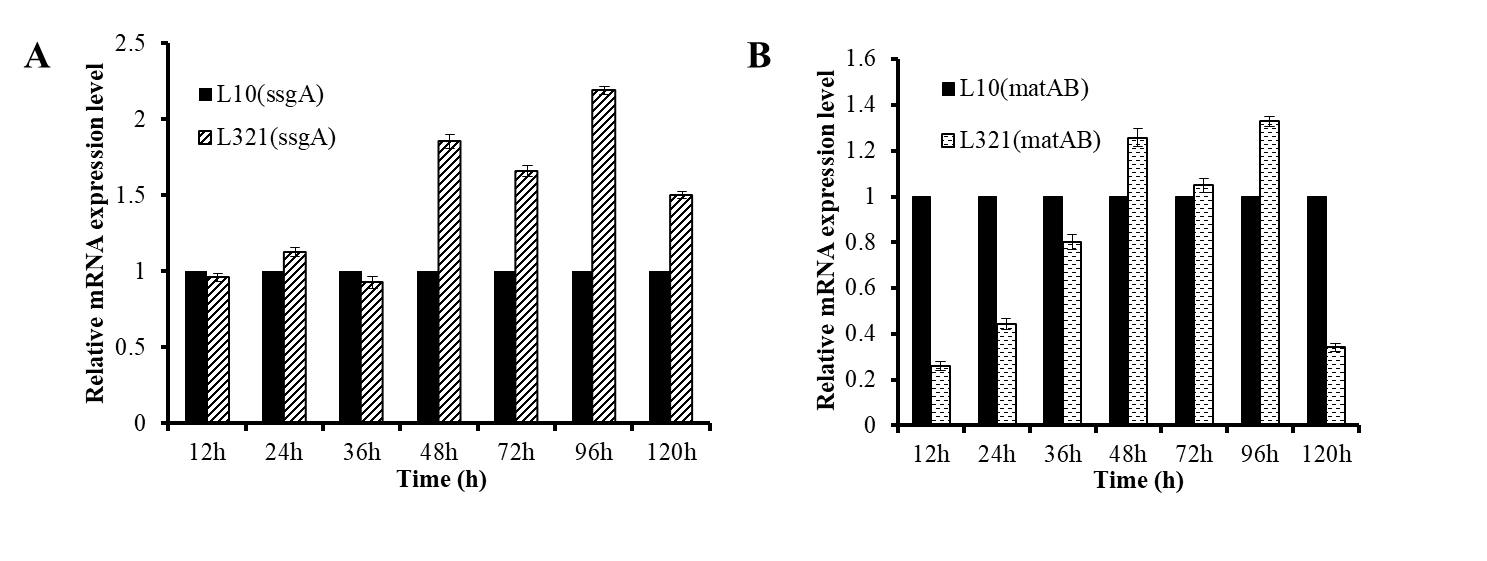

Supplement: Supplementary file 5 — Additional file 5. Relative mRNA expression level of morphogenesis in S. chattanoogensis L10 and L321. Transcription analysis of morphogenesis ssgA, clsA and matAB was carried out by qRT-PCR. clsA gene barely expressed, so the data were not shown. The transcription of sigma factor hrdB gene was assessed as an internal control. The expression level of theses morphogenesis in L10 is set as 1. The error bars represent standard deviations of the means of triplicate samples. [file 12934_2019_1055_MOESM5_ESM.docx]

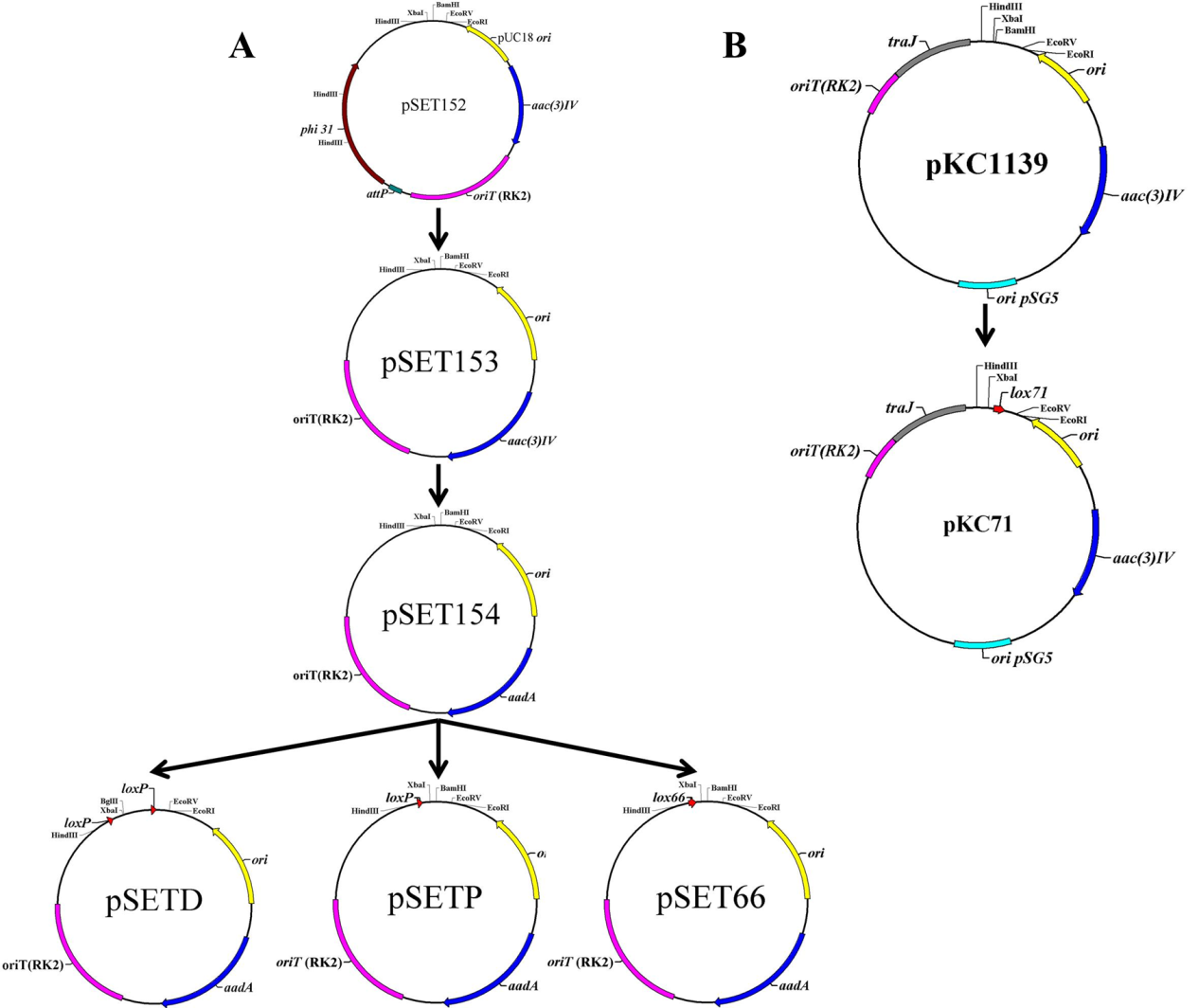

Supplement: Supplementary file 9 — Additional file 9. Schematic diagrams of universal plasmids pSETD, pSETP, pSET66 (A) and pKC71 (B). [file 12934_2019_1055_MOESM9_ESM.docx]
