# Supplementary material for: Rational construction of genome-reduced and high-efficient industrial Streptomyces chassis based on multiple comparative genomic approaches
Source: Microb Cell Fact. 2019 Jan 28;18:16. doi: 10.1186/s12934-019-1055-7 (PMC6348691; doi:10.1186/s12934-019-1055-7)
Supplement: Supplementary file 6 — Additional file 6. Table S2 shows plasmids used in this study and short description. [file 12934_2019_1055_MOESM6_ESM.docx]

| Plasmid | Characterization | Source |
| --- | --- | --- |
| pTA2 | TA-cloning vector, *bla* | Toyobo |
| pUG66 | Template plasmid containing two *loxP* sites, *bla* | [[1](#_ENREF_1)] |
| pSET152 | Integrative plasmid, *acc(3)IV*, phi 31 integrase, *attP* | [[2](#_ENREF_2)] |
| pKC1139 | Temperature-sensitive plasmid, pSG5 *ori*, *acc(3)IV* | [[3](#_ENREF_3)] |
| pL97 | pIJ101 replicon, ermE promoter, *acc(3)IV* | [[4](#_ENREF_4)] |
| pL99 | nitAp promoter induced by ε-caprolactam, pIJ101 replicon, *acc(3)IV* | [[4](#_ENREF_4)] |
| pL100 | pIJ101 replicon, ermE promoter, egfp, *acc(3)IV* | [[4](#_ENREF_4)] |
| pTOS | Integrative plasmid, VWB integrase, *acc(3)IV*, rox sites | [[5](#_ENREF_5)] |
| pALCre | Expression vector of Cre enzyme induced by thiostrepton(tsr), hyg, pIJ101 *ori* | [[5](#_ENREF_5)] |
| pMM1 | pSET152 derivative integrative plasmid harboring actinorhodin biosynthetic gene cluster | Yemin Wang, unpublished |
| pSET153 | pSET152 derivative deleting integrase gene by HindIII digestion and self-ligation, suicide vector without replicon of *Streptomyces* | This study |
| pSET154 | pSET153 derivative replacing *aac(3)IV* with *aadA* from pIJ779 | This study |
| pSETD | pSET154 derivative harboring two *loxP* sites at same direction flanking MCS | This study |
| pSETP | pSET154 derivative harboring MCS and one *loxP* site | This study |
| pSET66 | pSET154 derivative harboring MCS and *lox66* site | This study |
| pSATDF | pSETD derivative harboring two *loxP* sites and a 3Kb homologous fragment | This study |
| pSATPR | pSETP derivative harboring one *loxP* site and a 2.6 Kb homologous fragment | This study |
| pSLR | pSET66 derivative harboring one *loxP* site and a 2.0Kb homologous fragment for deleting 1.3Mb non-essential gene region | This study |
| pSRR | pSET66 derivative harboring one *loxP* site and a 2.0Kb homologous fragment for deleting 0.7Mb non-essential gene region | This study |
| pKC71 | pKC1139 derivative harboring MCS and *lox71* site | This study |
| pKCLF | pKC71 derivative harboring two homologous fragments flanking *lox71* site deleting 1.3Mb non-essential gene region | This study |
| pKCRF | pKC71 derivative harboring two homologous fragments flanking *lox71* site deleting 0.7Mb non-essential gene region | This study |
| pNitCre | pL99 derivative harboring codon optimized *Cre* gene from pALCre in MCS | This study |
| pTOSE | pTOS derivative harboring ermE promoter | This study |
| pTEindC | pTOSE derivative harboring a single-module non-ribosomal peptide synthase encoding gene *indC* | This study |

1. Gueldener U, Heinisch J, Koehler GJ, Voss D, Hegemann JH. A second set of loxP marker cassettes for Cre-mediated multiple gene knockouts in budding yeast. Nucleic Acids Res*.* 2002; 30:e23.

2. Anzai Y, Iizaka Y, Li W, Idemoto N, Tsukada S, Koike K, Kinoshita K, Kato F. Production of rosamicin derivatives in *Micromonospora rosaria* by introduction of d-mycinose biosynthetic gene with phi31-derived integration vector pSET152. J Ind Microbiol Biotechnol*.* 2009; 36:1013-1021.

3. Bierman M, Logan R, Obrien K, Seno ET, Rao RN, Schoner BE. Plasmid Cloning Vectors for the Conjugal Transfer of DNA from *Escherichia-Coli* to *Streptomyces Spp*. Gene*.* 1992; 116:43-49.

4. Sun N, Wang ZB, Wu HP, Mao XM, Li YQ. Construction of over-expression shuttle vectors in *Streptomyces*. Ann Microbiol*.* 2012; 62:1541-1546.

5. Herrmann S, Siegl T, Luzhetska M, Petzke L, Jilg C, Welle E, Erb A, Leadlay PF, Bechthold A, Luzhetskyy A. Site-Specific Recombination Strategies for Engineering Actinomycete Genomes. Appl Environ Microbiol*.* 2012; 78:1804-1812.
