# Supplementary material for: Rational construction of genome-reduced and high-efficient industrial Streptomyces chassis based on multiple comparative genomic approaches
Source: Microb Cell Fact. 2019 Jan 28;18:16. doi: 10.1186/s12934-019-1055-7 (PMC6348691; doi:10.1186/s12934-019-1055-7)
Supplement: Supplementary file 7 — Additional file 7. Table S3 shows strains used in this study and short description. [file 12934_2019_1055_MOESM7_ESM.docx]

| Strains | Characterization | Source |
| --- | --- | --- |
| *E.coli* |  |  |
| TG1 | Cloning host, K12 *(lac^-^pro), supE, thi, hsd5/F'[traD36, proAB, lacIq, lacZ M15]* | Novagen |
| DH10B | Cloning host, F^-^ *mcrA ∆(mrr^-^hsdRMS^-^mcrBC) φ80lacZ∆M15 ∆lacX74 recA1 endA1 araD139 ∆(ara, leu)7697 galE15 galK* λ^-^ *rpsL nupG* | Novagen |
| ET12567/pUZ8002 | Conjugation host, F^-^ *dam^-^13::Tn9 dcm^-^6 hsdM hsdR zjj^-^202::Tn10 recF143 galK2 galT22 ara^-^14 lacY1 xyl^-^5 leuB6 thi^-^1 tonA31 rpsL136 hisG4 tsx^-^78 mtl^-^1 glnV44* | [[1](#_ENREF_1)] |
| *S. chattanoogensis* |  |  |
| L10 | Wild type | [[2](#_ENREF_2)] |
| L102 | L10 derivative harboring pIJ8668-ermEp-egfp | This study |
| L103 | L10 derivative harboring pTEindC | This study |
| L104 | L10 derivative harboring pMM1 | This study |
| L320 | L10 derivative Δ499650bp—1841266bp | This study |
| L321 | L10 derivative Δ7994797bp—8731201bp | This study |
| L322 | L321 derivative harboring pIJ8668-*ermEp*-*egfp* | This study |
| L323 | L321 derivative harboring pTEindC | This study |
| L324 | L321 derivative harboring pMM1 | This study |

1. Flett F, Mersinias V, Smith CP. High efficiency intergeneric conjugal transfer of plasmid DNA from Escherichia coli to methyl DNA-restricting streptomycetes. FEMS Microbiol Lett*.* 1997; 155:223-229.

2. Du YL, Chen SF, Cheng LY, Shen XL, Tian Y, Li YQ. Identification of a Novel *Streptomyces chattanoogensis* L10 and Enhancing Its Natamycin Production by Overexpressing Positive Regulator ScnRII. J Microbiol*.* 2009; 47:506-513.
