# Supplementary material for: Rational construction of genome-reduced and high-efficient industrial Streptomyces chassis based on multiple comparative genomic approaches
Source: Microb Cell Fact. 2019 Jan 28;18:16. doi: 10.1186/s12934-019-1055-7 (PMC6348691; doi:10.1186/s12934-019-1055-7)
Supplement: Supplementary file 8 — Additional file 8. Table S4 shows primers used in this study and short description. [file 12934_2019_1055_MOESM8_ESM.docx]

| Primer | Sequence (5’-3’)*^a^* | Description |
| --- | --- | --- |
| aadA-F | GAGCTCcttcggaatagttatttgccgac | Amplification spectinomycin resistance gene (*aadA*) from pIJ779 inserted into pSET153 |
| aadA-R | GAGCTCagttcgtacagtctatgcctc |  |
| loxP-F1 | AAGCTTcgtacgctgcaggt | Amplification DNA fragment containing *loxP* site from pUG66 inserted into pSET154 |
| loxP-R1 | AGATCTctagacctaataacttcg |  |
| loxP-F2 | AGATCTgagctctcgagaacccttaat | Amplification DNA fragment containing loxP site from pUG66 inserted into pSET154 |
| loxP-R2 | GAATTCgatatcacctaataacttc |  |
| lox66-F | AAGCTTATAACTTCGTATAGCATACATTATACGAACGGTA | Primer pairs annealing to form lox66 site fragment inserted into pSET154 |
| lox66-R | TCTAGATACCGTTCGTATAATGTATGCTATACGAAGTTAT |  |
| lox71-F | TCTAGATACCGTTCGTATAGCATACATTATACGAAGTTAT | Primer pairs annealing to form lox71 site fragment inserted into pKC1139 |
| lox71-R | GATATCATAACTTCGTATAATGTATGCTATACGAACGGTA |  |
| ATD-F | TCTAGAGGAGTTCGAGGGTTCGGGTGT | Amplification 3 Kb homologous arm inserted into pSETD |
| ATD-R | AGATCTGCGGATCTGGTGCTGGACG |  |
| ATP-F | GATATCCCCTCCGTCCATTCTTTGA | Amplification 2.6 Kb homologous arm inserted into pSETP |
| ATP-R | GAATTCTTCGTCGTGCCTTGGTGA |  |
| LR-F | GATATCcagggccacccgcgagaccctg | Amplification 2.0 Kb homologous arm inserted into pSET66 |
| LR-R | GAATTCgccggacagccaggagcgcag |  |
| LF-F1 | AAGCTTgcgttgccagagccgcgtgg | Amplification 1.0 Kb homologous arm inserted into pKC71 |
| LF-R1 | TCTAGAcgaggagcttggacagcgtc |  |
| LF-F2 | GATATCcctagacacctccaccacac | Amplification 1.0 Kb homologous arm inserted into pKC71 |
| LF-R2 | GAATTCgggtcggcgtgagtgctagg |  |
| RR-F | GATATCctcgcgtgctggggcgccgg | 2.0 Kb homologous arm amplification pSET66 |
| RR-R | GAATTCcgcagctcgtcggccaggcg |  |
| RF-F1 | AAGCTTccaaggagatcgaggaccgc | Amplification 1.0 Kb homologous arm inserted into pKC71 |
| RF-R1 | TCTAGAgggcggccagcctgacgtac |  |
| RF-F2 | GATATCtcgccgccgacggcgggtcc | Amplification 1.0 Kb homologous arm inserted into pKC71 |
| RF-R2 | GAATTCgcaggccatgacggcgctgc |  |
| Cre-F | CATATGtccaacctgctgaccgtccac | Amplification *Cre* gene inserted into pL99 |
| Cre-R | GGATCCtcagtcgccgtcttccagcagg |  |
| indC-F | CATATGAGCACCAGCACGCCGCC | Amplification indC gene inserted into pTOSE |
| indC-R | TCTAGAACTAGGCGAAGAGGTCCAAGG |  |
| ssgA-F | GCGATATGCCCGTGACCTG | qRT-PCR primer for *ssgA* |
| ssgA-R | GCCCACTTGGAGGCTGATG |  |
| clsA-F | CTCGTCTGGTTCGGGTTCCT | qRT-PCR primer for *clsA* |
| clsA-R | GGTGCGCTTCATGCTGTGC |  |
| matAB-F | GAGATGGCAGCGCATGGAG | qRT-PCR primer for *matAB* |
| matAB-R | GCCGGAAATCGTTGTGGG |  |

^a^ Restriction sites were underlined
